# Supplementary material for: Regulatory Diversification of INDEHISCENT in the Capsella Genus Directs Variation in Fruit Morphology
Source: Curr Biol. 2019 Mar 18;29(6):1038–1046.e4. doi: 10.1016/j.cub.2019.01.057 (PMC6428689; doi:10.1016/j.cub.2019.01.057)
Supplement: Document S1. Figures S1–S3 [file mmc1.pdf]

Current Biology, Volume *29*

Supplemental Information

Regulatory Diversification of *INDEHISCENT* in the *Capsella* Genus Directs Variation in Fruit Morphology

Yang Dong, Friederike Jantzen, Nicola Stacey, Łukasz Łangowski, Laila Moubayidin, Jan Šimura, Karin Ljung, and Lars Østergaard

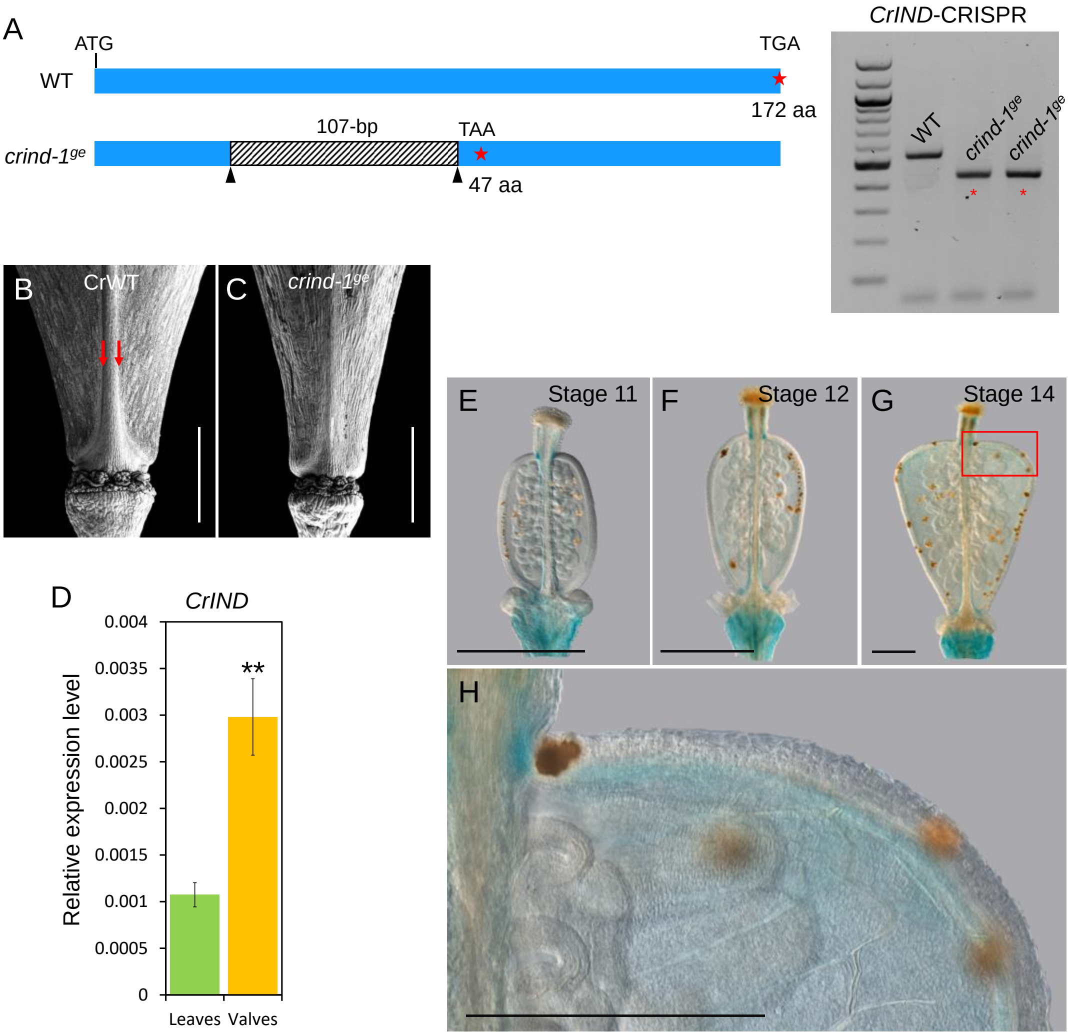

Figure S1. Genotyping of *CrIND* CRISPR line and expression analysis of *CrIND*, Related to Figure 1.

(A) Genotyping of *CrIND* CRISPR lines identified an allele with 107-bp deletion which generate a premature stop codon generating a truncated protein of 47 amino acids in length. (B and C) SEM pictures of basal part of the fruit of CrWT (B) and *crind-1^{ge}* showing the development of valve margins wild type (red arrows). Scale bars, 400 µm. (D) Comparative gene expression analysis of *CrIND* between leaves and fruit valves. Error bars represent SD of three biological replicates. **p<0.01 (Student's *t*-test). (E-H) Expression pattern of *CrIND* during fruit development with *pCrIND:GUS* line. (H) show the enlarged picture of the region outlined with red box in (G). Scale bars, 100 µm.

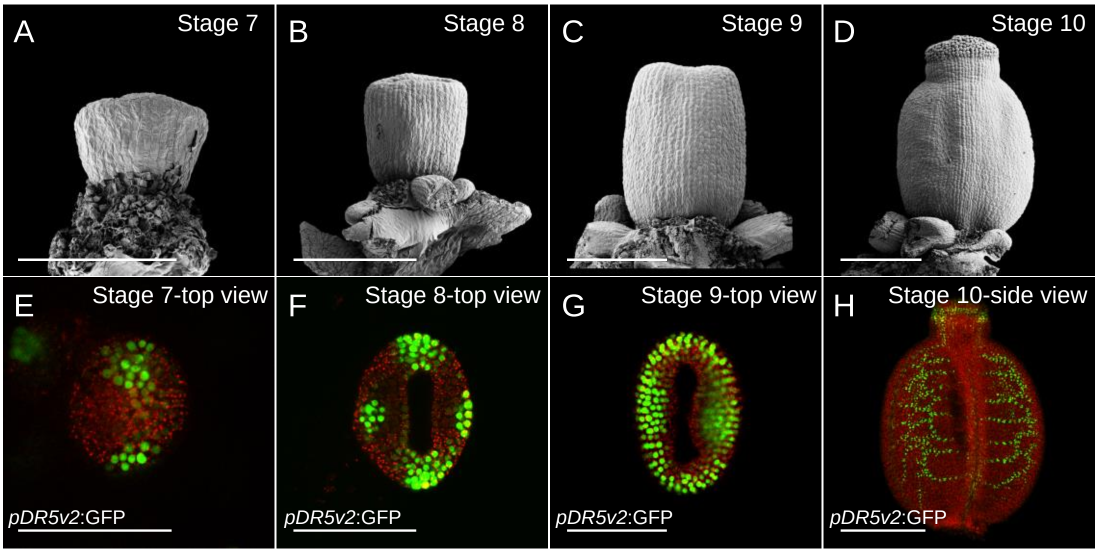

Figure S2. Ontogeny and auxin signaling dynamic during early gynoecium development in *Capsella*, Related to Figure 2.

(A-D) Ontogenetic analysis of the gynoecium of different developmental stages. (E-H) Auxin signaling in gynoecia of different developmental stages as shown by *pDR5v2:GFP*. Scale bars (A-H), 100 μm.

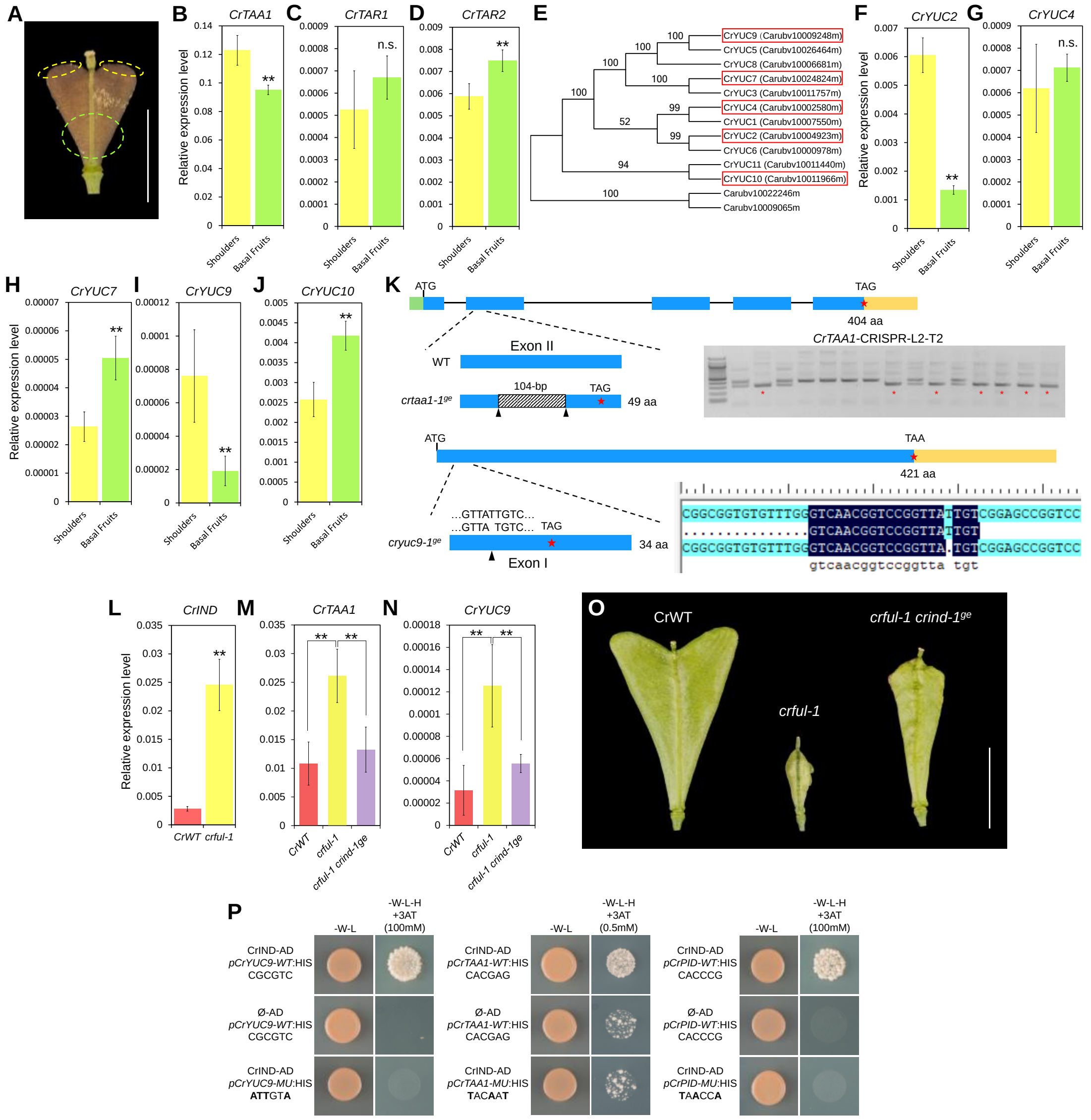

Figure S3. Gene expression analysis of the candidate genes and relationship between *CrIND* and auxin biosynthesis genes, Related to Figure 3.

(A) Graphic view of the fruit tissues sampled for expression analysis. Scale bar, 2 mm. (B-D) Gene expression analysis of the three *Capsella TAA1/TAR* genes compared between tissues sampled as shown in panel A. (E) Neighbor-joining tree of proteins encoded by the 11 *YUC* genes from the *Capsella* genome, bootstrap values over 50% (1,000 replicates) are indicated for each branch. (F-J) Gene expression analysis of five genes belonging to the *YUCCA* family compared between tissues sampled as shown in panel A. (K) Genotyping of the *CrTAA1* and *CrYUC9* CRISPR mutants identified a *crtaa1-1*^{ge} allele with 104-bp deletion in the second exon and a *cryuc9-1*^{ge} allele with a one-base pair deletion in the first exon. (L) Expression analysis of *CrIND* in the whole fruit of CrWT and *crful-1*. (M) and (N) Expression analysis of *CrTAA1* (M) and *CrYUC9* (N) in the whole fruit of CrWT, *crful-1* and *crful-1 crind-1*^{ge} stage-17 fruits. (O) Whole-mount images of fruits from CrWT, *crful-1* and *crful-1 crind-1*^{ge} at stage 17. Scale bar, 5 mm. (P) Yeast-one-hybrid analysis of the interaction of CrIND protein with the variant E box found in the promoters of *CrTAA1* and *CrYUC9*. *CrPID* was used as a positive control. Error bars in (B-D, F-J, L-N) represent SD of three biological replicates. ***p*<0.01 (Student's *t*-test).

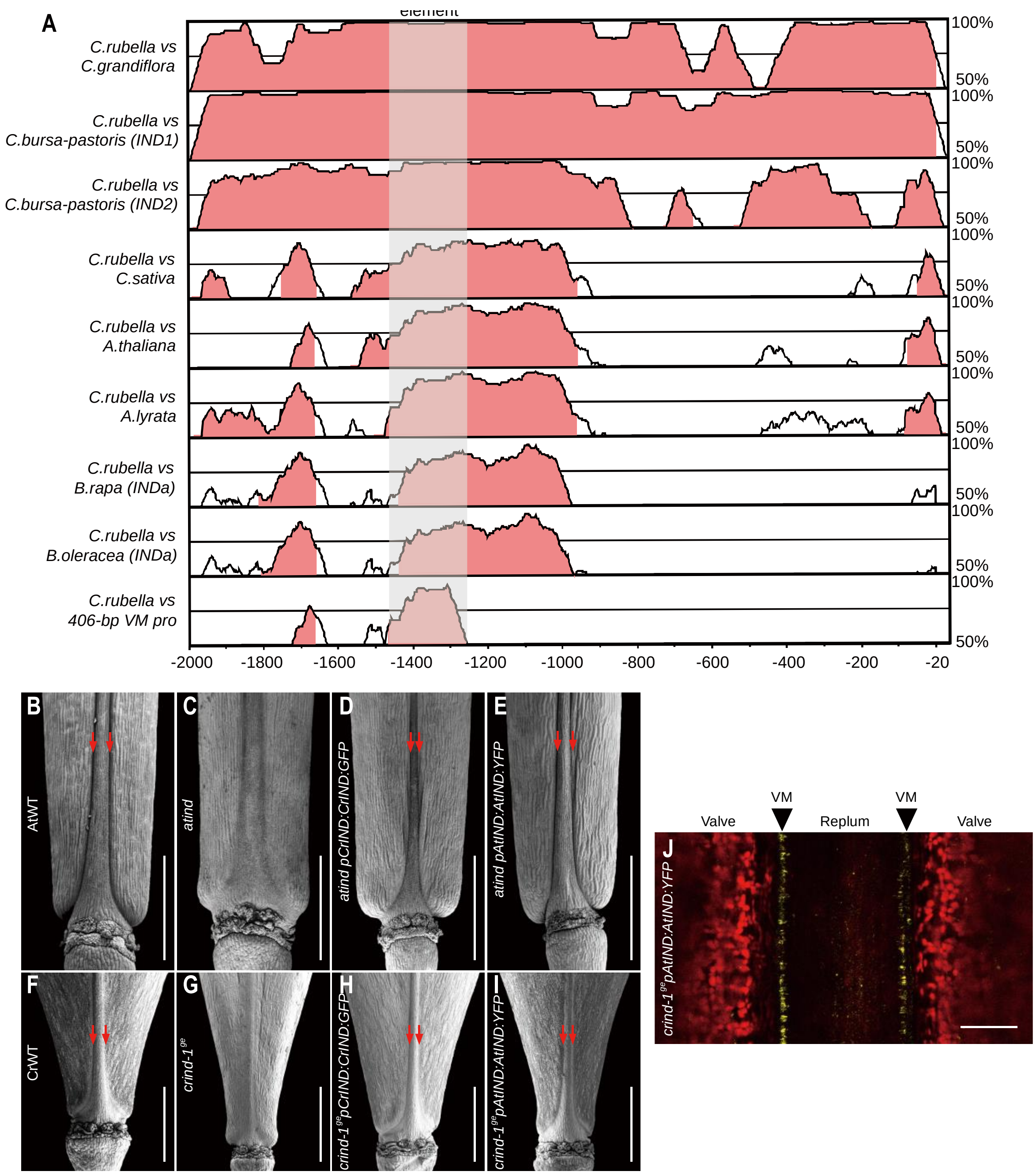

Figure S4. Regulatory divergence in the *CrIND* promoter explains the expression expansion of *CrIND* from the valve margin into the valves, Related to Figure 4.

(A) Phylogenetic shadowing using mVISTA of a ~2.1 kb promoter region upstream of the translational start site of the *IND* gene with pairwise alignments of sequences from *Capsella rubella* with *C. grandiflora, C. bursa-pastoris* (two paralogues), *Camelina sativa*, *Arabidopsis thaliana*, *A. lyrata*, *Brassica rapa*, *B. oleracea* and the 406-bp valve margin element identified previously. Position of the Valve Margin element (VM element) is indicated by a shaded area. (B-E) SEM images of basal fruit of AtWT (B), *atind* (C), *atind pCrIND:CrIND:GFP* (D) and *atind pAtIND:AtIND:YFP* (E). Red arrows in (B), (D) and (E) indicate the valve margin. (F-I) SEM images of basal fruit (8DPA) of CrWT (F), *crind-1*^{ge} (G), *crind-1*^{ge} *pCrIND:CrIND:GFP* (H), *crind-1*^{ge} *pAtIND:AtIND:CFP* (I). (J) Confocal images of fruits of *crind-1*^{ge} *pAtIND:AtIND:YFP* , YFP expression was only detected in the valve margin (VM). Red arrows in (F), (H) and (I) indicate the valve margin. Scale bars, 100 μm (J), 400 μm (B-I).
