# Supplementary material for: Regulatory Diversification of INDEHISCENT in the Capsella Genus Directs Variation in Fruit Morphology
Source: Curr Biol. 2019 Mar 18;29(6):1038–1046.e4. doi: 10.1016/j.cub.2019.01.057 (PMC6428689; doi:10.1016/j.cub.2019.01.057)
Supplement: Methods S1. Primers Used in This Study, Related to STAR Methods [file mmc2.docx]

**Methods S1**

| **Primer** | **Sequence (5’ to 3’)** | **Experiment** |
| --- | --- | --- |
| *pDR5rev*-F | GAATTCGTCGACGGTATCGCA | Reporter Plasmid Construction |
| *pDR5rev*-R | ATCCATGGTGTTATATCTCCTTGGATC | Reporter Plasmid Construction |
| *pCrIND*-GUS-F | CCTCTAGAGTCGACCTGCAGGATGCCTAAATTAGCTTTTGATG | Reporter Plasmid Construction |
| *pCrIND*-GUS-R | CTCAGATCTACCATGGGCTGTTCTTGGTTGGATCTGCTT | Reporter Plasmid Construction |
| *CrIND:*GFP-F | ATCCTCTAGAGTCGACGATGCCTAAATTAGCTTTTGATG | Reporter Plasmid Construction |
| *CrIND:*GFP-R | GTCAGATCTACCATGGAGGTTTGGGAGTTGTGGTAAT | Reporter Plasmid Construction |
| *pCrTAA1*-GUS-F | CCTCTAGAGTCGACCTGCAGTGAGTGAATGAGATAGTTTG | Reporter Plasmid Construction |
| *pCrTAA1*-GUS-R | CTCAGATCTACCATGGCTTCGTTCTTCTTCCTCTTC | Reporter Plasmid Construction |
| *PCrYUC9*-GUS-F | CCTCTAGAGTCGACCTGCAGACAAATTGATAATTCGAAGATTA | Reporter Plasmid Construction |
| *PCrYUC9*-GUS-R | CTCAGATCTACCATGGTTGTTTTTTAGAGTGGGTTTTGAAC | Reporter Plasmid Construction |
| *iaaL*-F | CCTCTAGAGTCGACCTGCAGATGACTGCCTACGATATGGAAAAG | Plasmid Construction |
| *iaaL*-R | CTCAGATCTACCATGGTCAGTTTCGGCGGTCGATGATG | Plasmid Construction |
| *iaaM*-F | CCTCTAGAGTCGACCTGCAGATGTCAGCTTCACCTCTCCT | Plasmid Construction |
| *iaaM*-R | CTCAGATCTACCATGGCTAATTTCTAGTGCGGTAGTTATATC | Plasmid Construction |
| *CrYUC2*-rt-F | CCAATATTGGCTCATCCTCAAC | Expression Analysis |
| *CrYUC2*-rt-R | ATGTCACAATTAAAGAGTTAGGAAC | Expression Analysis |
| *CrYUC4*-rt-F | GTTGGAAAGGAGAGAATGGAC | Expression Analysis |
| *CrYUC4*-rt-R | AAACATTCCTACAACTACTCAACG | Expression Analysis |
| *CrYUC7*-rt-F | CGGGAATAGCTAAGTTTGGTCC | Expression Analysis |
| *CrYUC7*-rt-R | TTCTTCTCTATCCCAATCTCTGCC | Expression Analysis |

| **Primer** | **Sequence (5’ to 3’)** | **Experiment** |
| --- | --- | --- |
| *CrYUC9*-rt-F | GTTAGGGATGGATTAACAGAAAAGG | Expression Analysis |
| *CrYUC9*-rt-R | TTACAAAACGCCCATACAGGTG | Expression Analysis |
| *CrYUC10*-rt-F | AACTGTCAAAAAGATCCGCGAT | Expression Analysis |
| *CrYUC10*-rt-R | CCTCTAACCAATTGCAGACAGAA | Expression Analysis |
| *CrTAA1*-rt-F | AGTCGTGAAGATGTTTTCAAC | Expression Analysis |
| *CrTAA1*-rt-R | GATAAACTTTAGGGTAAAAGCTAG | Expression Analysis |
| *CrTAR1*-rt-F | GGAAAGAGATCTCGGAAGCCTC | Expression Analysis |
| *CrTAR1*-rt-R | AGTCTATGGAGAAAGACGTCATAGT | Expression Analysis |
| *CrTAR2*-rt-F | TGTGAGAAGTTTCTAAGAGAGG | Expression Analysis |
| *CrTAR2*-rt-R | TTAATTACAAAGTTGAATTAGAGG | Expression Analysis |
| *CrIAA5*-rt-F | GGATGCTTGCGGGAGACGTTCC | Expression Analysis |
| *CrIAA5*-rt-R | GTGCCGCGTGCTGGTCATCCTC | Expression Analysis |
| *CrGH3.3*-rt-F | ACCAGAACGACGTCGTTGAG | Expression Analysis |
| *CrGH3.3*-rt-R | TCAGACAAAAGGAGCTAGGGAG | Expression Analysis |
| *CrSAUR16*-rt-F | AACATCTGAACATATCATCCACG | Expression Analysis |
| *CrSAUR16*-rt-R | CAACTCAAACAAATTAACCGTAATC | Expression Analysis |
| *CrACTIN7*-rt-F | GGAAACATCGTTCTCAGTGGT | Expression Analysis |
| *CrACTIN7*-rt-R | CTTGATCTTCATGCTGCTAGGT | Expression Analysis |
| *CrUBQ10*-rt-F | ATGCAGATCTTCGTTAAGACTC | Expression Analysis |
| *CrUBQ10*-rt-R | CAAGGTACGGCCATCCTCCAAC | Expression Analysis |
| *CrIND*-gRNA1 | CCTCCTCAGCTCATGTTGGATCC | CRISPR/Cas9 Gene Editing |
| *CrIND*-gRNA2 | CCCGTAGACATCGACCCTTCGAC | CRISPR/Cas9 Gene Editing |

| **Primer** | **Sequence (5’ to 3’)** | **Experiment** |
| --- | --- | --- |
| *CrTAA1*-gRNA1 | CAACGGCTTACGAAGAATAC | CRISPR/Cas9 Gene Editing |
| *CrTAA1*-gRNA2 | GCGACATGATCAACTTGTGT | CRISPR/Cas9 Gene Editing |
| *CrYUC9*-gRNA1 | GTCAACGGTCCGGTTATTGT | CRISPR/Cas9 Gene Editing |
| *CrYUC9*-gRNA2 | CAGATTGCATAGCTTCACTG | CRISPR/Cas9 Gene Editing |
| *CrIND*-geno-F | AAAGTCTAGTGCGGGTCTCG | Genotyping |
| *CrIND*-geno-R | CATCAATAAGCAATAGAGAT | Genotyping |
| *CrTAA1*-geno-F | ATCATAGGCTCAAAACAAATCG | Genotyping |
| *CrTAA1*-geno-R | GTTTAGTGGTGGGGACTTACGG | Genotyping |
| *CrYUC9*-geno-F | GAATATGTTTAGACTAACGGCAAG | Genotyping |
| *CrYUC9*-geno-R | GCATTTTCGGTAATTGACAG | Genotyping |
| *CrTAA1*-ChIP-F | AAGCCTCTCTGATGGTATTAGC | ChIP Assay |
| *CrTAA1*-ChIP-R | TTTTACTTCCTTCTTTGGCTTC | ChIP Assay |
| *CrYUC9*-ChIP-F | TTACATGTAAGAAACAAGTTTG | ChIP Assay |
| *CrYUC9*-ChIP-R | ATTAATGATCGATATAATGAAG | ChIP Assay |
| *CrPID*-ChIP-F | TTTCAAGCACGTGACAACGTCC | ChIP Assay |
| *CrPID*-ChIP-R | ACAGACACCTTTTTTGACTGTGGG | ChIP Assay |
| *CrYUC9*-Y1H-WT | ATTTATGATAGATCATCGCGTCACATTCATCGCGTCACATTCATCGCGTCACATTCATCGCGTCACATGCAATCATGCAT | Yeast-one-hybrid |
| *CrYUC9*-Y1H-MU | ATTTATGATAGATCATATTGTAACATTCATATTGTAACATTCATATTGTAACATTCATATTGTAACATGCAATCATGCAT | Yeast-one-hybrid |

| **Primer** | **Sequence (5’ to 3’)** | **Experiment** |
| --- | --- | --- |
| *CrYUC9*-Y1H-WT | ATTTATGATAGATCATCGCGTCACATTCATCGCGTCACATTCATCGCGTCACATTCATCGCGTCACATGCAATCATGCAT | Yeast-one-hybrid |
| *CrYUC9*-Y1H-MU | ATTTATGATAGATCATATTGTAACATTCATATTGTAACATTCATATTGTAACATTCATATTGTAACATGCAATCATGCAT | Yeast-one-hybrid |
| *CrTAA1*-Y1H-WT | CTCAATACTTATATTTCACGAGTTGAATTTCACGAGTTGAATTTCACGAGTTGAATTTCACGAGTTGATTGGTCACTACA | Yeast-one-hybrid |
| *CrTAA1*-Y1H-MU | CTCAATACTTATATTTTACAATTTGAATTTTACAATTTGAATTTTACAATTTGAATTTTACAATTTGATTGGTCACTACA | Yeast-one-hybrid |
| *CrPID*-Y1H-WT | CCTCCTCTCCTTTCCGCACCCGTTGATCCGCACCCGTTGATCCGCACCCGTTGATCCGCACCCGTTGAAAAAGTACAATC | Yeast-one-hybrid |
| *CrPID*-Y1H-MU | CCTCCTCTCCTTTCCGTAACCATTGATCCGTAACCATTGATCCGTAACCATTGATCCGTAACCATTGAAAAAGTACAATC | Yeast-one-hybrid |
