# Supplementary material for: Regulatory Diversification of INDEHISCENT in the Capsella Genus Directs Variation in Fruit Morphology
Source: Curr Biol. 2019 Mar 18;29(6):1038–1046.e4. doi: 10.1016/j.cub.2019.01.057 (PMC6428689; doi:10.1016/j.cub.2019.01.057)
Supplement: Document S2. Article plus Supplemental Information [file mmc3.pdf]

Report

Current Biology

Regulatory Diversification of *INDEHISCENT* in the *Capsella* Genus Directs Variation in Fruit Morphology

Highlights

- Fruit-shape defect observed in *crind* mutant is rescued by exogenous auxin application
- Auxin dynamics are perturbed in *crind* mutant
- *CrIND* directly controls expression of auxin-biosynthesis genes in fruit valves
- *CrIND* regulatory sequences contribute to the morphological novelty of *Capsella* fruits

Authors

Yang Dong, Friederike Jantzen, Nicola Stacey, ..., Jan Šimura, Karin Ljung, Lars Østergaard

Correspondence

lars.ostergaard@jic.ac.uk

In Brief

Dong et al. demonstrate that diversification of *INDEHISCENT* expression in the *Capsella* genus has contributed to morphological changes of the heart-shaped fruit compared to the cylindrical *Arabidopsis* fruit and that *INDEHISCENT* mediates its effect via localized activation of auxin-biosynthesis genes.

Dong et al., 2019, Current Biology *29*, 1038–1046
March 18, 2019 © 2019 The Author(s). Published by Elsevier Ltd.
https://doi.org/10.1016/j.cub.2019.01.057

Cell Press
Current Biology
Report

Regulatory Diversification of *INDEHISCENT* in the *Capsella* Genus Directs Variation in Fruit Morphology

Yang Dong,[1] Friederike Jantzen,[1,3] Nicola Stacey,[1] Łukasz Łangowski,[1,4] Laila Moubayidin,[1] Jan Šimura,[2] Karin Ljung,[2] and Lars Østergaard[1,5,*]
[1]Crop Genetics Department, John Innes Centre, Norwich NR4 7UH, UK
[2]Umeå Plant Science Centre, Department of Forest Genetics and Plant Physiology, Swedish University of Agricultural Sciences, SE-901 83 Umeå, Sweden
[3]Present address: Institute for Biochemistry and Biology, University of Potsdam, D-14476 Potsdam-Golm, Germany
[4]Present address: Brandon Products Ltd., Centrepoint, Tralee, Co., Kerry, Ireland
[5]Lead Contact
*Correspondence: lars.ostergaard@jic.ac.uk
https://doi.org/10.1016/j.cub.2019.01.057

SUMMARY

Evolution of gene-regulatory sequences is considered the primary driver of morphological variation [1–3]. In animals, the diversity of body plans between distantly related phyla is due to the differential expression patterns of conserved "toolkit" genes [4]. In plants, variation in expression domains similarly underlie most of the reported diversity of organ shape both in natural evolution and in the domestication of crops [5–9]. The heart-shaped fruit from members of the *Capsella* genus is a morphological novelty that has evolved after *Capsella* diverged from *Arabidopsis* ∼8 mya [10]. Comparative studies of fruit growth in *Capsella* and *Arabidopsis* revealed that the difference in shape is caused by local control of anisotropic growth [11]. Here, we show that sequence variation in regulatory domains of the fruit-tissue identity gene, *INDEHISCENT* (*IND*), is responsible for expansion of its expression domain in the heart-shaped fruits from *Capsella rubella*. We demonstrate that expression of this *CrIND* gene in the apical part of the valves in *Capsella* contributes to the heart-shaped appearance. While studies on morphological diversity have revealed the importance of *cis*-regulatory sequence evolution, few examples exist where the downstream effects of such variation have been characterized in detail. We describe here how CrIND exerts its function on *Capsella* fruit shape by binding sequence elements of auxin biosynthesis genes to activate their expression and ensure auxin accumulation into highly localized maxima in the fruit valves. Thus, our data provide a direct link between changes in expression pattern and altered hormone homeostasis in the evolution of morphological novelty.

RESULTS AND DISCUSSION

The *INDEHISCENT* Gene Controls Fruit Shape in *Capsella rubella*

The Brassicaceae family is characterized by a remarkable diversity in fruit shape between different genera [12]. Even so, the overall tissue composition of Brassicaceae fruits is highly conserved with valves enclosing the seeds, a replum in the center of the fruit, and valve margins that form the border between the valves and the replum [13]. The overall fruit-shape diversity is primarily due to variation in valve morphology. For example, fruits from the *Capsella* genus develop valves that are extended at the apical end, giving them a heart-shaped appearance [11, 12] (Figure 1A and 1B). This shape is unique to fruits from *Capsella* species; fruits from the closest relative, *Camelina*, are spherical, while *Arabidopsis* produce cylindrically shaped fruits [10, 14] (Figure 1A). Comparative studies between the development of fruits from *Capsella* and other Brassicaceae therefore provides an excellent system to study the molecular mechanisms underlying morphological changes [15].

In a previous study, we demonstrated that the master regulator of valve development, *FRUITFULL* (*FUL*), has conserved functions in both *Arabidopsis* and *Capsella* based on highly similar *ful* loss-of-function phenotypes [11]. In a continued effort to test for diversity of function between known key regulators of fruit development in the Brassicaceae family, we created a knockout line of the *Capsella rubella IND* gene (*CrIND*) using CRISPR/Cas9, leading to a 107-bp deletion within the coding region (Figures 1B and S1A). This mutant allele was named *crind-1^{ge}*, where "ge" stands for "genome editing" according to guidelines recently published for *Marchantia* gene nomenclature [16]. In agreement with the function of *IND* in both *Arabidopsis* and *Brassica* valve-margin formation [17, 18], the *crind-1^{ge}* mutant fruits do not form valve margins and are as a consequence completely indehiscent (Figures S1B and S1C). Additionally, mature *crind-1^{ge}* fruits exhibit a reduction in the development of the shoulders (measured as a shoulder index, Figure 1K) compared to wild-type, indicating that *CrIND* has a role in *Capsella* fruit-shape formation (Figure 1A, 1B, and 1L). In *Arabidopsis*, the *IND* gene (*AtIND*) is specifically expressed in the valve

 © 2019 The Author(s). Published by Elsevier Ltd.
This is an open access article under the CC BY license (http://creativecommons.org/licenses/by/4.0/).

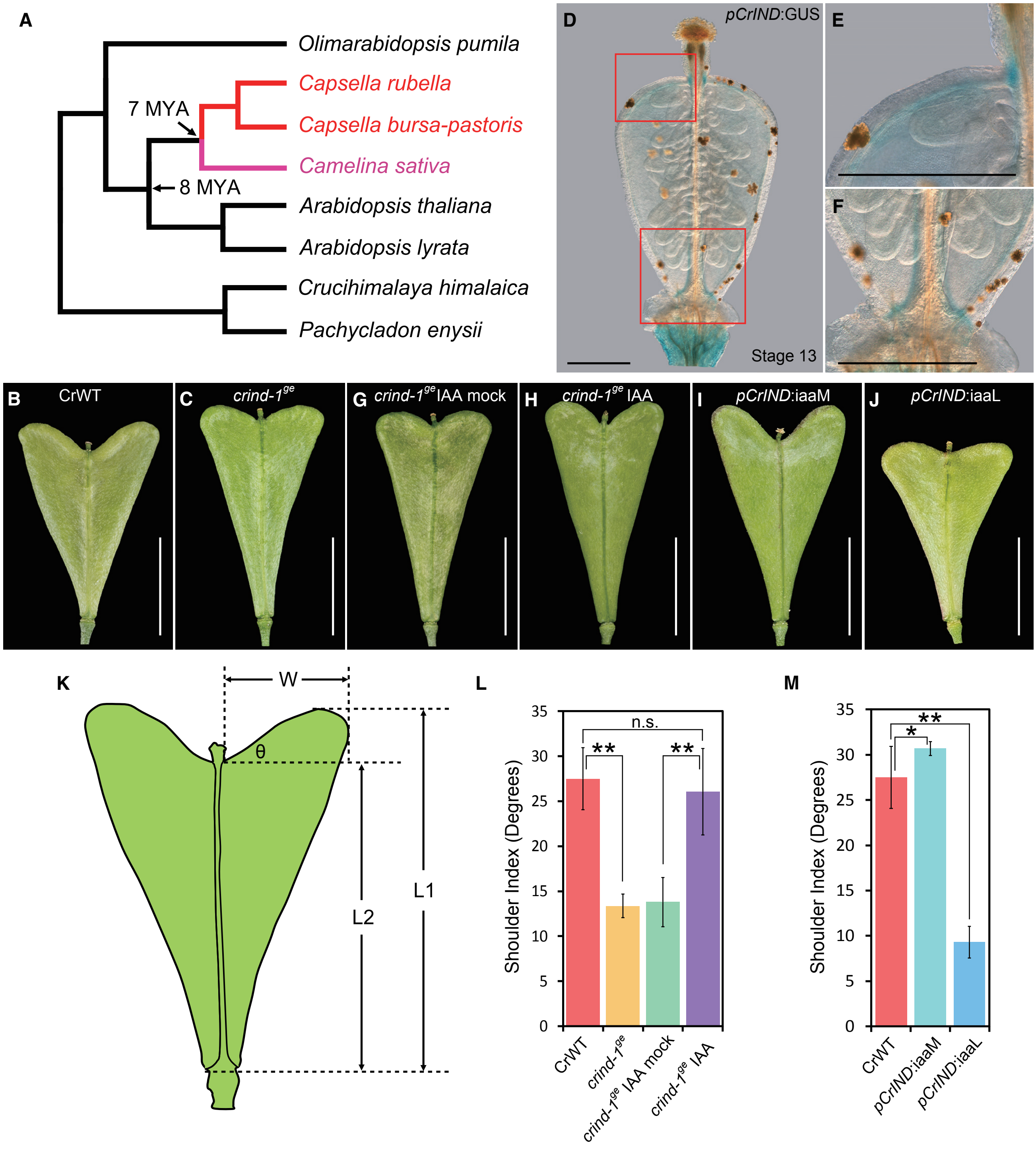

Figure 1. Effect of *CrIND* and Manipulation of Auxin Levels on *Capsella* Fruit Shape

(A) A simplified phylogeny of *Capsella* and its close relatives according to [10]. The heart-shaped fruit from *Capsella* (red) shares a common ancestor of *Camelina* (magenta), which develops spherical siliques. The black branches in the phylogeny represent the species with cylindrical fruit.

(B and C) Fruit morphology of CrWT (B) and *crind-1^{ge}* (C) at developmental stage 17.

(D–F) Expression pattern of *CrIND* during fruit development with *pCrIND:GUS* line. (E) and (F) show enlarged pictures of the regions outlined with red box in (D) with valve expression (E) and valve margin expression (F), respectively.

(G and H) Fruit morphology of *crind-1^{ge}* after mock (G) or IAA (H) treatment at stage 17.

(I and J) Fruit morphology of *pCrIND:iaaM* (I) and *pCrIND:iaaL* (J) at stage 17.

(K) Schematic drawing to illustrate the shoulder index calculation.

(legend continued on next page)

margins after fertilization of the ovary [17, 19]. We tested whether the role of *CrIND* in valve-shape formation could be due to a change in expression pattern compared to *AtIND* in *Arabidopsis* or could be a result of differential growth caused by loss of valve-margin tissue. In support of the former, we detected expression of *CrIND* in *Capsella* valves by quantitative RT-PCR (qRT-PCR) (Figure S1D). To examine more specifically the *CrIND* expression pattern in the *Capsella* valves, we constructed a *pCrIND:GUS* reporter and found GUS signal in the apical parts in addition to the signal in the valve margin (Figures 1D–1F and S1E–S1H). These data suggest that *CrIND* affects fruit-shape formation cell autonomously due to an expansion of its expression domain in the developing shoulders.

The function of *IND* in both valve-margin specification and earlier during gynoecium development has been closely associated with auxin dynamics [19, 20]. Therefore, we investigated whether a link to auxin could also be established for *CrIND* in fruit-shape formation. We found that application of exogenous auxin (indole-3-acetic acid or IAA) to the apex of *crind-1^{ge}* mutant fruit rescued the growth defect observed in the valves (Figure 1G, 1H, and 1L). Moreover, expression of a bacterial auxin biosynthesis gene, *iaaM* [21], under the *CrIND* promoter in a wild-type background led to shoulders that were extended further than in wild-type (Figure 1I and 1M). In contrast, depleting free IAA in the same domain by expressing the *iaaL* gene [22] under control of the *CrIND* promoter significantly reduces the shoulder index of the heart-shaped fruits (Figures 1J and 1M).

CrIND Is Required to Maintain Auxin Homeostasis in *Capsella* Fruit Valves

The auxin-signaling reporter *pDR5:revGFP* has previously been used to map the dynamics of auxin during *Arabidopsis* gynoecium development [20, 23]. It has been demonstrated that auxin is required to mediate a symmetry transition in the apical style through its tightly controlled accumulation in specific auxin maxima [20]. Here, we used two modified DR5 reporters, *pDR5v2:GFP* and *pDR5v2:GUS* [24] transformed into *C. rubella*. The *pDR5v2:GFP* reporter mimicked the pattern in the style observed in *Arabidopsis* during the early developmental stages (Figure S2A-H) [20]. In contrast to *Arabidopsis*, DR5 signal was also observed in the vascular tissue of the *Capsella* valves at developmental stage 10 (stages defined in [13, 25]) (Figure S2H). At later stages, when the *Capsella* gynoecium develops from an oblate spheroid (flat disc) into an emerging heart shape [11] (Figures 2A–2D), the DR5v2 reporter is clearly observed in the apical part of the valves with very specific maxima in the shoulders (Figures 2F–2I). Interestingly, this expression pattern is reduced in stage-14 fruits of *crind-1^{ge}*, when the defect in shoulder development has emerged (Figures 2E and 2J). The reduction of auxin signaling in the *crind-1^{ge}* fruit shoulders correlated with qRT-PCR data showing that expression of three different auxin-responsive genes is significantly reduced in fruit shoulders from *crind-1^{ge}* compared to wild-type (Figures 2K–2M). In contrast, expression of the house-keeping gene, *CrACTIN7*, was not significantly different (Figure 2N). In line with this observation, direct measurements of both the predominant natural auxin, indole-3-acetic acid (IAA), and its precursor, indole-3-pyruvate (IPA), show a significant reduction in the shoulders of *crind-1^{ge}* mutant (Figures 2O and 2P). Together, these results show that *CrIND* mediates its function on *Capsella* fruit shape by local control of auxin dynamics in the shoulders of the valves.

CrIND Directly Regulates Auxin Biosynthesis Genes to Control *Capsella* Fruit Shape

The rescue of the *crind-1^{ge}* valve-shape phenotype by application of auxin (Figures 1G, 1H, and 1L) combined with the reduced auxin levels measured in the *crind-1^{ge}* mutant (Figures 2O and 2P) suggest that CrIND is involved in controlling auxin biosynthesis. We first tested if inhibition of auxin biosynthesis affects the development of wild-type fruits. Therefore, we applied inhibitors of the two steps leading to the synthesis of IAA from tryptophan. The first step leading to IPA is catalyzed by enzymes belonging to the TAA1/TAR family [26] and can be inhibited by *L*-Kynurenine [27], whereas the second step from IPA to IAA is mediated by members of the YUCCA family [28], which are inhibited by Yucasin [29]. Treatment with *L*-Kynurenine and Yucasin led to fruits with strongly reduced shoulder indices (Figures 3A–3C and 3N). We next carried out a comparative expression analysis of members of the *TAA1/TAR* and *YUCCA* gene families in *Capsella* to screen for genes that are more highly expressed in the shoulders relative to the base (Figure S3A). For the three members of the TAA1/TAR family, only TAA1 showed this pattern (Figures S3B–S3D), and a *pCrTAA1:GUS* reporter line exhibited very specific expression in the developing shoulders (Figures 3F–3I), suggesting highly localized auxin biosynthesis. The YUCCA family consists of 11 genes that can be divided into five clades based on their sequence identity (Figure S3E). We carried out qRT-PCR for representatives of each clade—namely, *CrYUC2*, *CrYUC4*, *CrYUC7*, *CrYUC9*, and *CrYUC10*. Of these, expression of *CrYUC2* and *CrYUC9* was significantly higher in the shoulders compared to the base of the fruit (Figures S3F–S3J). GUS-reporter lines were developed for *CrYUC2* and *CrYUC9*; however, a signal could only be detected for the *pCrYUC9:GUS* reporter. Similar to *CrTAA1*, *pCrYUC9:GUS* exhibited specific expression in the shoulders, compatible with a role in mediating auxin biosynthesis at these sites (Figures 3J–3M). Therefore, expression of both *CrTAA1* and *CrYUC9* overlap with expression of the DR5v2 reporter line (Figures 2G–2I). In agreement with the recognized importance for local auxin biosynthesis throughout plant development [30], these data suggest a specific role for the TAA/YUC auxin-biosynthesis pathway in generating a highly specific auxin maximum at the valve apices. To test if *CrTAA1* and *CrYUC9* are required for fruit-shape formation in *Capsella*, we generated knockout lines

(L) Shoulder index measurements of fruits from CrWT, *crind-1^{ge}*, and *crind-1^{ge}* ± IAA treatment. Error bars represent SD of 30 individual fruits.
(M) Shoulder index measurements of fruits from WT, *pCrIND:iaaM*, and *pCrIND:iaaM* plants. Error bars represent SD of 30 individual fruits.
Scale bars represent 5 mm for (B), (C), and (G)–(J) and 100 μm for (D)–(F). $^{**}p < 0.01$ (Student's t test) in (L) and (M).
See also Figure S1.

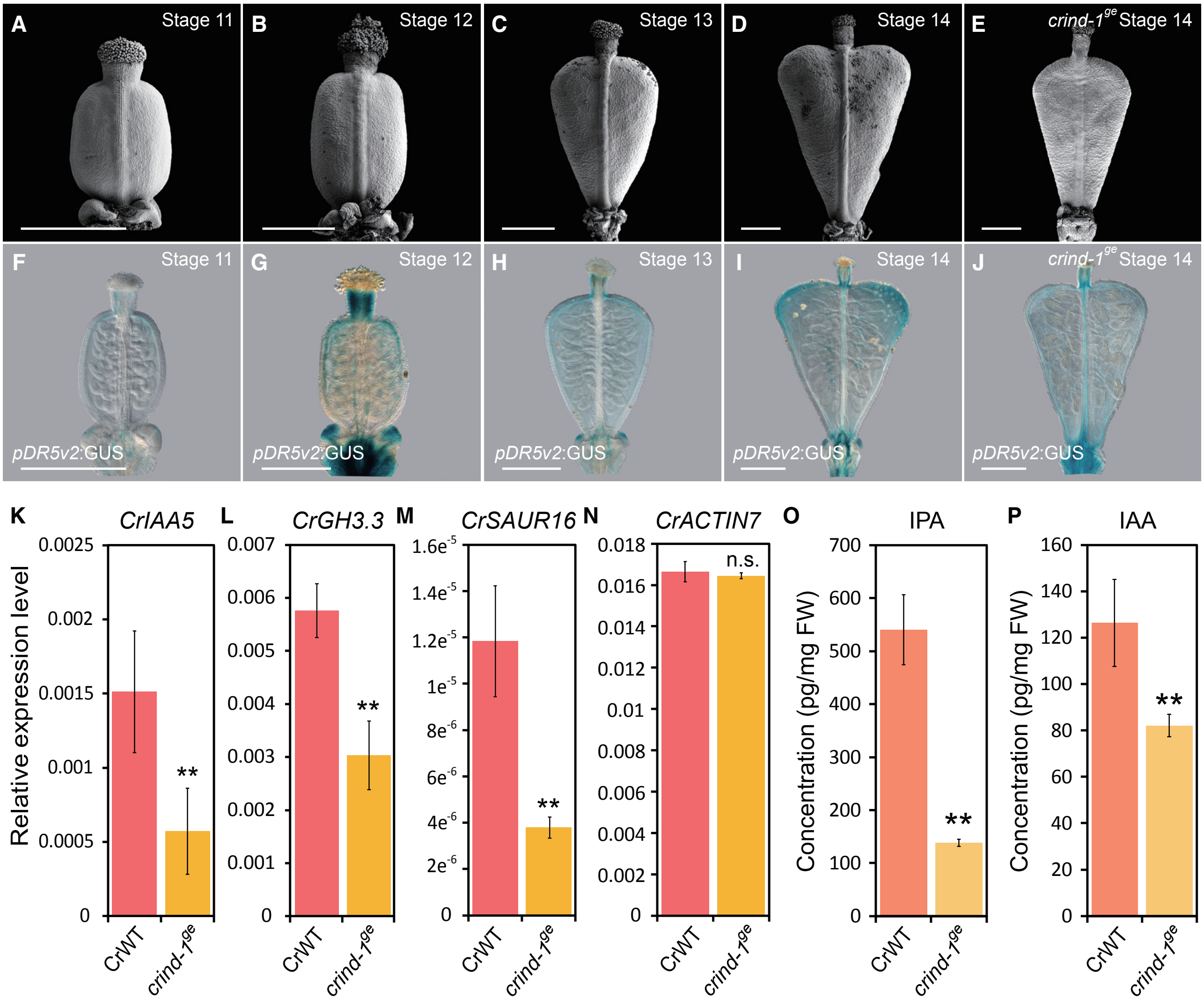

Figure 2. Auxin Dynamics during *Capsella* Fruit Development in Wild-Type and the *crind* Mutant

(A–E) SEM images of fruits from CrWT at developmental stages 11 (A), 12 (B), 13 (C), and 14 (D) and from *crind-1^{ge}* at stage 14 (E).

(F–J) Auxin signaling visualized by *pDR5v2:GUS* in CrWT fruit of developmental stages 11 (F), 12 (G), 13 (H), and 14 (I) and in the *crind-1^{ge}* mutant at stage 14 (J).

(K–N) Expression analysis by qRT-PCR of *CrIAA5* (K), *CrGH3.3* (L), *CrSAUR16* (M), and *CrACTIN7* (N) in the fruit shoulders of WT and *crind-1^{ge}* at stage 14. Error bars represent SD of three biological replicates.

(O and P) Measurements of IPA (O) and IAA (P) in fruit shoulders of WT and *crind-1^{ge}* stage-14 fruits. Error bars represent SD of three biological replicates.

Scale bars represent 150 μm (A–J). **$p < 0.01$ (Student's t test) in (K–P).

See also Figure S2.

using CRISPR/Cas9, leading to a 104-bp deletion in Exon II of *CrTAA1* (*crtaa1-1^{ge}*) and a 1-bp deletion in Exon I of *CrYUC9* (*cryuc9-1^{ge}*) (Figure S3K). Both mutations resulted in reduced valve growth (Figures 3D, 3E, and 3N) similar to the treatments with the auxin-biosynthesis inhibitors. However, the *cryuc9-1^{ge}* mutant fruits were less severely affected in shoulder growth compared to *crtaa1-1^{ge}*, which may be due to residual activity of CrYUC2 in the *cryuc9-1^{ge}* background.

To test whether *CrIND* regulates *CrTAA1* and *CrYUC9*, we performed qRT-PCR using RNA extracted from wild-type and *crind-1^{ge}* mutant fruits and found significantly reduced levels of both *CrTAA1* and *CrYUC9* mRNA in the mutant (Figures 3O and 3P). FUL in *Arabidopsis* (AtFUL) is a repressor of *AtIND*, excluding *AtIND* expression from the valves and restricting it to the valve margins. As a consequence, the *AtIND* expression level is elevated in *atful* mutant fruits [17, 31]. Similarly, in fruits from the *Capsella crful-1* mutant [11], *CrIND* expression was upregulated (Figure S3L). In agreement with CrIND positively regulating the expression of *CrTAA1* and *CrYUC9*, we found that these genes were upregulated in *crful-1*, while this effect was abolished in the *crful-1 crind-1^{ge}* double mutant (Figures S3M and S3N).

The *crful-1* mutant fruits have a severe growth defect similar to that reported for *heegeri*, which is a natural variant of the tetraploid *C. bursa-pastoris* [11, 32]. This clearly shows that other factors than *CrIND* are involved in determining the heart shape. As in

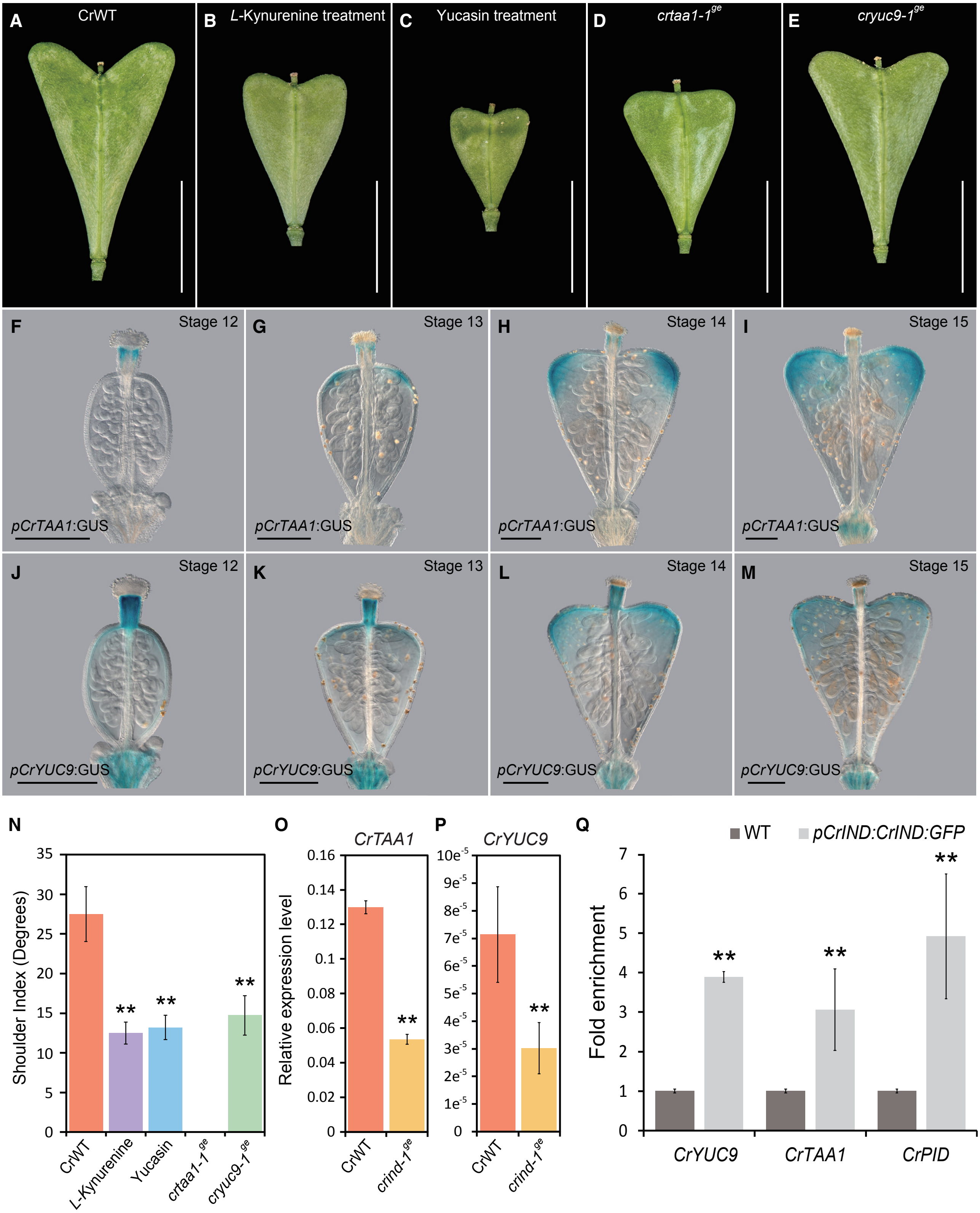

Figure 3. CrIND-Induced Expression of Auxin Biosynthesis Gene

(A–C) Whole-mount images showing morphologies of CrWT fruits 8 DPA of mock-treated (A), treated with *L*-Kynurenine (B) and Yucasin (C).

(D and E) Whole-mount images of *crtaa1-1*^{ge} and *cryuc9-1*^{ge}.

(legend continued on next page)

Arabidopsis, loss of *IND* leads to a significant rescue of the growth defects of the *crful-1* mutant. However, this is not accompanied by the development of shoulders, which supports that CrIND is required for the local induction of *CrTAA1* and *CrYUC9* expression (Figure S3O).

In previous studies, we have found that IND directly regulates genes that affect auxin dynamics such as the protein kinase genes *PINOID* (*PID*) and *WAG2* by binding to a variant "E-box" (CACGCG) in the regulatory regions [19, 33]. An analysis of the promoter regions of *CrTAA1* and *CrYUC9* identified potential CrIND recognition sites (CACGAG for *CrTAA1* and CGCGTC for *CrYUC9*). Using *crind-1^{ge}* plants complemented with *pCrIND:CrIND:GFP* (Figure 4G), we performed chromatin immunoprecipitation (ChIP) on fruit tissue and showed that CrIND directly interacts with promoter regions of both *CrTAA1* and *CrYUC9* (Figure 3Q). The specific binding of CrIND protein to the identified elements was further tested by yeast one-hybrid (Figure S3P). Taken together, these results suggest that rather than initiating shoulder formation per se, CrIND promotes growth after shoulder initiation by inducing localized expression of auxin biosynthesis genes. We hypothesize that establishment of localized auxin maxima at the shoulder tips provides polarity and thus stimulates anisotropic growth in their direction. This is similar to the effects of auxin maxima observed in other developmental contexts such as lateral root growth and gynoecium development [20, 23].

Regulatory Divergence in the *IND* Genes of *Capsella* and *Arabidopsis* Contributes to the Morphological Differences in Fruit Shape

The heart-shaped fruit is unique to the *Capsella* genus in the Brassicaceae family and evolved after *Camelina* and *Capsella* diverged ~7 mya [10] (Figure 1A). In both animals and plants, morphological novelties most often arise from mutations in regulatory sequences that alter gene expression patterns rather than in protein-encoding regions [1–4, 34, 35]. The expanded expression of *CrIND* in the fruit valves of *Capsella* strongly suggests that the *CrIND* promoter has diverged from other Brassicaceae *IND* sequences. Previously, we found that the valve margin-specific expression of *AtIND* was governed by sequence contained in a 406-bp promoter element [18]. We extracted ~2.1 kb regulatory sequences of nine Brassicaceae *IND* genes, including four from the *Capsella* genus. A phylogenetic shadowing analysis was carried out using the mVISTA software [36] to assess regional conservation across species. In this analysis, pairwise comparison of the *CrIND* promoter sequence against *IND* sequences from the other eight species revealed a highly conserved region, which includes the region required for valve-margin expression (Figure S4A). Interestingly, this analysis also revealed large regions of the promoter where the *Capsella* sequences are highly conserved but diverge from other species. Conceivably, these *Capsella*-specific regions contain elements that have allowed for the expanded *IND* expression in *Capsella* (Figure S4A).

While the expanded expression of *CrIND* in the valves compared to *IND* in *Arabidopsis* could be due to changes in the regulatory sequence of the *CrIND* gene itself, it is also possible that *CrIND* expression in the valve apices is caused by differential expression of an upstream regulator. To distinguish between these two possibilities, we first transformed the *atind-2* mutant from *Arabidopsis* with a *pCrIND:CrIND:GFP* construct. Resulting transgenic lines were fully dehiscent, demonstrating that the *pCrIND:CrIND:GFP* gene complemented the indehiscence phenotype of the *atind-2* mutant similarly to the effect of the *pAtIND:AtIND:YFP* construct (Figures 4A–4D and S4B–S4E). However, while the *atind-2 pAtIND:AtIND:YFP* fruits had a wild-type shape, fruits from *atind-2 pCrIND:CrIND:GFP* plants have abnormal apices where valve growth expands above the style (Figures 4C and 4D). Conversely, the *pAtIND:AtIND:YFP* construct only restored the dehiscence defect of the *crind-1^{ge}* mutant, but not the shape change (Figures 4E–4H and S4F–S4I). This is supported by the expression pattern of the *pAtIND:AtIND:YFP*, which was undetectable in the valves but observed in valve margins (Figure S4J). In contrast, the *pCrIND:CrIND:GFP* construct complemented both defects (Figures 4G and S4H). These results show that expansion of *CrIND* expression into the valves in *Capsella* is due to changes in *cis*-regulatory sequences in the *CrIND* gene itself and that this has contributed to the change in fruit shape between these two genera. It is interesting to speculate that the modified expression pattern of *CrIND* may have led to novel genetic interactions such as described for KNOX genes in leaf development [9], thereby facilitating the recognition of the auxin biosynthesis target genes in the valves.

Concluding Remarks

In animals, changes in *cis*-regulatory elements of otherwise conserved "toolkit" genes is the primary driver of morphological evolution [2, 3, 37]. A similar pattern is emerging in plants, where modifications of regulatory sequences have been revealed as the major determinant of developmental variation both during domestication and natural evolution [5–7]. Even so, examples have also been reported where changes in protein-coding sequence are either fully or partly responsible for the evolution of morphological diversity [38, 39]. The work described here directly links changes in expression domain of

(F–M) Expression pattern of *CrTAA1* and *CrYUC9* shown by GUS staining of the *pCrTAA1:GUS* (F–I) and *pCrYUC9:GUS* (J–M) reporter lines at developmental stages 12 (F and J), 13 (G and K), 14 (H and L) and 15 (I and M).

(N) Shoulder indices of fruits from CrWT, *L*-Kynurenine treatment, Yucasin-treatment, *crtaa1-1^{ge}*, and *cryuc9-1^{ge}* fruits. N.D. indicate not determinable. Error bars represent SD of 30 individual fruits.

(O and P) Expression analysis of *CrTAA1* (N) and *CrYUC9* (O) in the fruit shoulders of CrWT and *crind-1^{ge}* stage-14 fruits. Error bars represent SD of three biological replicates.

(Q) Chromatin Immuno-Precipitation (ChIP) analysis of *CrIND* associated with the *CrTAA1* and *CrYUC9* promoter. The *CrPINOID* (*CrPID*) was used as a positive control, the potential E boxes bound by CrIND are shown below each gene. Error bars represent SD of three biological replicates.

Scale bars represent 5 mm (A–E) and 150 μm (F–M). **p < 0.01 (Student's t test) in (N–R).

See also Figure S3.

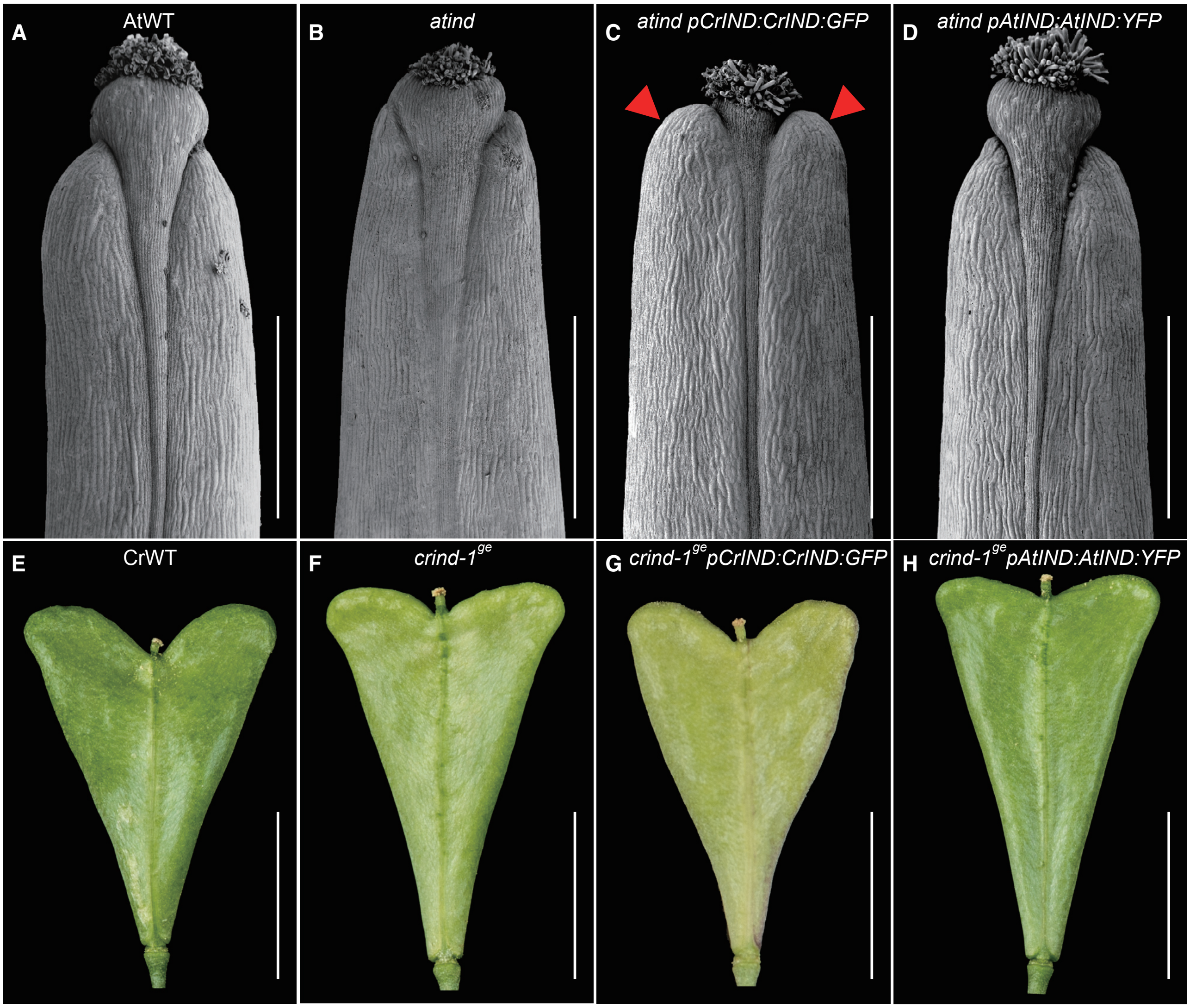

Figure 4. Morphological Effects of *AtIND* and *CrIND* Expression

(A–D) SEM images of the apex of stage-17 fruits of AtWT (A), *atind-2* (B), *atind-2 pCrIND:CrIND:GFP* (C), and *atind-2 pAtIND:AtIND:YFP* (D). The red triangles in (D) indicate the expanded apical growth of the valve tips.

(E–H) Fruit morphology at stage 17 of CrWT (E), *crind-1^{ge}* (F), *crind-1^{ge} pCrIND:CrIND:GFP* (G), and *crind-1^{ge} pAtIND:AtIND:YFP* (H).

Scale bars represent 5 mm (E–H) and 400 μm (A–D).

See also Figure S4.

a fruit-tissue-identity gene to effects on hormone homeostasis resulting in a morphological novelty. Given the stunning variation in fruit shape among members of the Brassicaceae family, it is possible that direct effects of gene-expression diversity on hormone dynamics is a common driver in the evolution of fruit-shape diversity.

STAR★METHODS

Detailed methods are provided in the online version of this paper and include the following:

- KEY RESOURCES TABLE
- CONTACT FOR REAGENT AND RESOURCE SHARING
- EXPERIMENTAL MODEL AND SUBJECT DETAILS
- METHOD DETAILS
  - Plasmids construction and plant transformation
  - Phenotyping and Microscopy
  - Chemical Treatment and Auxin Metabolite Quantification
  - RNA extraction and expression analysis
  - Chromatin immunoprecipitation and Yeast one-hybrid analysis
- QUANTIFICATION AND STATISTICAL ANALYSIS

SUPPLEMENTAL INFORMATION

Supplemental Information can be found with this article online at https://doi.org/10.1016/j.cub.2019.01.057.

ACKNOWLEDGMENTS

We are grateful to Heather Bland, Yuli Ding, Lauren Grubb, André Kuhn, Mikhaela Neequaye, Pauline Stephenson, Tongbing Su, and Billy Tasker-Brown for critically reading the manuscript and providing comments prior to submission. We thank Andrew Davies and Phil Robinson for photographic assistance. This study was supported by a grant from the Biotechnological and Biological Research Council (BBSRC) to L.Ø. (BB/P020747/1) and an Institute Strategic Programme Grant from the BBSRC to the John Innes Centre (BB/P013511/1). K.L. and J.Š. acknowledge the Knut and Alice Wallenberg Foundation (KAW), the Swedish Governmental Agency for Innovation Systems (VINNOVA), the Swedish Research Council (VR), and the Swedish Metabolomics Centre (https://www.swedishmetabolomicscentre.se/) for access to instrumentation.

AUTHOR CONTRIBUTIONS

Y.D. and L.Ø. designed the research. Y.D. performed most of the experiments with assistance from N.S. and scientific input from L.M. F.J. produced the *crind-1^{ge}* CRISPR allele, Ł.Ł. developed the *DR5v2 Capsella* lines, J.Š. and K.L. carried out IAA and IPA measurements, and L.Ø. did the phylogenetic shadowing analysis. Y.D. and L.Ø. outlined and wrote the manuscript, and L.Ø. supervised the project. All authors participated in the discussion of the data and in the production of the final version of the manuscript.

DECLARATION OF INTERESTS

The authors declare no competing interests.

Received: January 14, 2019
Revised: January 21, 2019
Accepted: January 23, 2019
Published: February 28, 2019

REFERENCES

1. Carroll, S.B. (2005). Evolution at two levels: on genes and form. PLoS Biol. *3*, e245.
2. Carroll, S.B. (2008). Evo-devo and an expanding evolutionary synthesis: a genetic theory of morphological evolution. Cell *134*, 25–36.
3. Wray, G.A. (2007). The evolutionary significance of cis-regulatory mutations. Nat. Rev. Genet. *8*, 206–216.
4. Carroll, S.B. (2000). Endless forms: the evolution of gene regulation and morphological diversity. Cell *101*, 577–580.
5. Wang, R.-L., Stec, A., Hey, J., Lukens, L., and Doebley, J. (1999). The limits of selection during maize domestication. Nature *398*, 236–239.
6. Konishi, S., Izawa, T., Lin, S.Y., Ebana, K., Fukuta, Y., Sasaki, T., and Yano, M. (2006). An SNP caused loss of seed shattering during rice domestication. Science *312*, 1392–1396.
7. Arnaud, N., Lawrenson, T., Østergaard, L., and Sablowski, R. (2011). The same regulatory point mutation changed seed-dispersal structures in evolution and domestication. Curr. Biol. *21*, 1215–1219.
8. Lemmon, Z.H., Bukowski, R., Sun, Q., and Doebley, J.F. (2014). The role of *cis* regulatory evolution in maize domestication. PLoS Genet. *10*, e1004745.
9. Rast-Somssich, M.I., Broholm, S., Jenkins, H., Canales, C., Vlad, D., Kwantes, M., Bilsborough, G., Dello Ioio, R., Ewing, R.M., Laufs, P., et al. (2015). Alternate wiring of a KNOXI genetic network underlies differences in leaf development of A. thaliana and C. hirsuta. Genes Dev. *29*, 2391–2404.
10. Hohmann, N., Wolf, E.M., Lysak, M.A., and Koch, M.A. (2015). A time-calibrated road map of Brassicaceae species radiation and evolutionary history. Plant Cell *27*, 2770–2784.
11. Eldridge, T., Łangowski, Ł., Stacey, N., Jantzen, F., Moubayidin, L., Sicard, A., Southam, P., Kennaway, R., Lenhard, M., Coen, E.S., and Østergaard, L. (2016). Fruit shape diversity in the Brassicaceae is generated by varying patterns of anisotropy. Development *143*, 3394–3406.
12. Łangowski, Ł., Stacey, N., and Østergaard, L. (2016). Diversification of fruit shape in the Brassicaceae family. Plant Reprod. *29*, 149–163.
13. Roeder, A.H.K., and Yanofsky, M.F. (2006). Fruit development in *Arabidopsis*. Arabidopsis Book *4*, e0075.
14. Bailey, C.D., Koch, M.A., Mayer, M., Mummenhoff, K., O'Kane, S.L., Jr., Warwick, S.I., Windham, M.D., and Al-Shehbaz, I.A. (2006). Toward a global phylogeny of the Brassicaceae. Mol. Biol. Evol. *23*, 2142–2160.
15. Sicard, A., and Lenhard, M. (2018). Capsella. Curr. Biol. *28*, R920–R921.
16. Bowman, J.L., Araki, T., Arteaga-Vazquez, M.A., Berger, F., Dolan, L., Haseloff, J., Ishizaki, K., Kyozuka, J., Lin, S.-S., Nagasaki, H., et al. (2016). The naming of names: Guidelines for gene nomenclature in *Marchantia*. Plant Cell Physiol. *57*, 257–261.
17. Liljegren, S.J., Roeder, A.H.K., Kempin, S.A., Gremski, K., Østergaard, L., Guimil, S., Reyes, D.K., and Yanofsky, M.F. (2004). Control of fruit patterning in *Arabidopsis* by INDEHISCENT. Cell *116*, 843–853.
18. Girin, T., Stephenson, P., Goldsack, C.M.P., Kempin, S.A., Perez, A., Pires, N., Sparrow, P.A., Wood, T.A., Yanofsky, M.F., and Østergaard, L. (2010). Brassicaceae INDEHISCENT genes specify valve margin cell fate and repress replum formation. Plant J. *63*, 329–338.
19. Sorefan, K., Girin, T., Liljegren, S.J., Ljung, K., Robles, P., Galván-Ampudia, C.S., Offringa, R., Friml, J., Yanofsky, M.F., and Østergaard, L. (2009). A regulated auxin minimum is required for seed dispersal in Arabidopsis. Nature *459*, 583–586.
20. Moubayidin, L., and Østergaard, L. (2014). Dynamic control of auxin distribution imposes a bilateral-to-radial symmetry switch during gynoecium development. Curr. Biol. *24*, 2743–2748.
21. Romano, C.P., Robson, P.R., Smith, H., Estelle, M., and Klee, H. (1995). Transgene-mediated auxin overproduction in *Arabidopsis*: hypocotyl elongation phenotype and interactions with the *hy6-1* hypocotyl elongation and *axr1* auxin-resistant mutants. Plant Mol. Biol. *27*, 1071–1083.
22. Romano, C.P., Hein, M.B., and Klee, H.J. (1991). Inactivation of auxin in tobacco transformed with the indoleacetic acid-lysine synthetase gene of *Pseudomonas savastanoi*. Genes Dev. *5*, 438–446.
23. Benková, E., Michniewicz, M., Sauer, M., Teichmann, T., Seifertová, D., Jürgens, G., and Friml, J. (2003). Local, efflux-dependent auxin gradients as a common module for plant organ formation. Cell *115*, 591–602.
24. Liao, C.Y., Smet, W., Brunoud, G., Yoshida, S., Vernoux, T., and Weijers, D. (2015). Reporters for sensitive and quantitative measurement of auxin response. Nat. Methods *12*, 207–210, 2, 210.
25. Smyth, D.R., Bowman, J.L., and Meyerowitz, E.M. (1990). Early flower development in *Arabidopsis*. Plant Cell *2*, 755–767.
26. Stepanova, A.N., Robertson-Hoyt, J., Yun, J., Benavente, L.M., Xie, D.Y., Dolezal, K., Schlereth, A., Jürgens, G., and Alonso, J.M. (2008). TAA1-mediated auxin biosynthesis is essential for hormone crosstalk and plant development. Cell *133*, 177–191.
27. He, W., Brumos, J., Li, H., Ji, Y., Ke, M., Gong, X., Zeng, Q., Li, W., Zhang, X., An, F., et al. (2011). A small-molecule screen identifies L-kynurenine as a competitive inhibitor of TAA1/TAR activity in ethylene-directed auxin biosynthesis and root growth in Arabidopsis. Plant Cell *23*, 3944–3960.
28. Zhao, Y., Christensen, S.K., Fankhauser, C., Cashman, J.R., Cohen, J.D., Weigel, D., and Chory, J. (2001). A role for flavin monooxygenase-like enzymes in auxin biosynthesis. Science *291*, 306–309.
29. Nishimura, T., Hayashi, K., Suzuki, H., Gyohda, A., Takaoka, C., Sakaguchi, Y., Matsumoto, S., Kasahara, H., Sakai, T., Kato, J., et al. (2014). Yucasin is a potent inhibitor of YUCCA, a key enzyme in auxin biosynthesis. Plant J. *77*, 352–366.
30. Zhao, Y. (2018). Essential roles of local auxin biosynthesis in plant development and in adaptation to environmental changes. Annu. Rev. Plant Biol. *69*, 417–435.
31. Ferrándiz, C., Liljegren, S.J., and Yanofsky, M.F. (2000). Negative regulation of the SHATTERPROOF genes by FRUITFULL during Arabidopsis fruit development. Science *289*, 436–438.
32. Shull, G.H. (1914). Duplicate genes for capsule-form in *Capsella bursa-pastoris*. Z. Abst. u. Vererbl. *12*, 97–149.

CellPress

33. Girin, T., Paicu, T., Stephenson, P., Fuentes, S., Körner, E., O'Brien, M., Sorefan, K., Wood, T.A., Balanzá, V., Ferrándiz, C., et al. (2011). INDEHISCENT and SPATULA interact to specify carpel and valve margin tissue and thus promote seed dispersal in *Arabidopsis*. Plant Cell *23*, 3641–3653.

34. Rebeiz, M., and Tsiantis, M. (2017). Enhancer evolution and the origins of morphological novelty. Curr. Opin. Genet. Dev. *45*, 115–123.

35. Jacob, F. (1977). Evolution and tinkering. Science *196*, 1161–1166.

36. Mayor, C., Brudno, M., Schwartz, J.R., Poliakov, A., Rubin, E.M., Frazer, K.A., Pachter, L.S., and Dubchak, I. (2000). VISTA : visualizing global DNA sequence alignments of arbitrary length. Bioinformatics *16*, 1046–1047.

37. Koshikawa, S., Giorgianni, M.W., Vaccaro, K., Kassner, V.A., Yoder, J.H., Werner, T., and Carroll, S.B. (2015). Gain of *cis*-regulatory activities underlies novel domains of *wingless* gene expression in *Drosophila*. Proc. Natl. Acad. Sci. USA *112*, 7524–7529.

38. Wang, H., Studer, A.J., Zhao, Q., Meeley, R., and Doebley, J.F. (2015). Evidence that the origin of naked kernels during maize domestication was caused by a single amino acid substitution in *tga1*. Genetics *200*, 965–974.

39. Vuolo, F., Mentink, R.A., Hajheidari, M., Bailey, C.D., Filatov, D.A., and Tsiantis, M. (2016). Coupled enhancer and coding sequence evolution of a homeobox gene shaped leaf diversity. Genes Dev. *30*, 2370–2375.

40. Simonini, S., Deb, J., Moubayidin, L., Stephenson, P., Valluru, M., Freire-Rios, A., Sorefan, K., Weijers, D., Friml, J., and Østergaard, L. (2016). A noncanonical auxin-sensing mechanism is required for organ morphogenesis in *Arabidopsis*. Genes Dev. *30*, 2286–2296.

41. Abràmoff, M.D., Magalhães, P.J., and Ram, S.J. (2004). Image processing with ImageJ. Biophoton. Int. *11*, 36–42.

42. Novák, O., Hényková, E., Sairanen, I., Kowalczyk, M., Pospíšil, T., and Ljung, K. (2012). Tissue-specific profiling of the Arabidopsis thaliana auxin metabolome. Plant J. *72*, 523–536.

STAR★METHODS

KEY RESOURCES TABLE

REAGENT or RESOURCE	SOURCE	IDENTIFIER
Antibodies		
Anti-GFP monoclonal antibody	Roche	11814460001
Bacterial Strains		
DH5-alpha competent *E. coli*	New England Biolabs	C29871
Agrobacterium tumefaciens strain LBA4404	N/A	N/A
Biological Samples		
Capsella rubella (22.5)	This paper	N/A
Arabidopsis thaliana (Col-0)	This paper	N/A
atind-2	[17]	N/A
atind-2 pAtIND:AtIND:YFP	[40]	N/A
crind-1^{ge}	This paper	N/A
crful-1	[11]	N/A
crful-1 crind-1^{ge}	This paper	N/A
crtaa1-1^{ge}	This paper	N/A
cryuc9-1^{ge}	This paper	N/A
Chemicals Peptides, and Recombinant Proteins		
Phusion High-Fidelity DNA polymerase	New England Biolabs	M0530L
DnaseI	QIAGEN	79254
In-Fusion Cloning Recombinase	Clontech	638909
Proteinase K	Invitrogen	59895
L-Kynurenine	Sigma-Aldrich	K8625
Yucasin	Carbosynth	FC1222381801
Indole-3-acetic acid (IAA)	Sigma-Aldrich	I5148
Chlorohydrate	Sigma-Aldrich	15307
DMSO	Sigma-Aldrich	D8418
Formaldehyde	Sigma-Aldrich	F8775
$K_3Fe(CN)_6$	Sigma-Aldrich	P8131
$K_4Fe(CN)_6$	Sigma-Aldrich	P9387
Triton X-100	Sigma-Aldrich	T8787
Cysteamine	Sigma-Aldrich	M9768
X-gluc	MELFORD	MB1121
Oligonucleotides		
A list of oligonucleotides is given in Methods S1		N/A
Other		
QIAprep Spin MiniPrep Kit	QIAGEN	27104
DNeasy Plant Mini Kit	QIAGEN	69104
QIAquick PCR Purification Kit	QIAGEN	28104
RNeasy Plant Mini Kit	QIAGEN	74104
Pierce Protein G Magnetic Beads	ThermoFisher	19958500
SuperScript™ IV First-Strand Synthesis System	ThermoFisher	18091050
SYBR Green JumpStart Taq ReadyMix	Sigma-Aldrich	S4438
Oasis HLB 1 cc Vac Cartridge	Waters	WAT094225
Recombinant DNA		
pDR5v2:GUS	This Paper	N/A
pDR5v2:GFP	This Paper	N/A

(*Continued on next page*)

Continued		
REAGENT or RESOURCE	SOURCE	IDENTIFIER
pCrIND:GUS	This Paper	N/A
pCrTAA1:GUS	This Paper	N/A
pCrYUC9:GUS	This Paper	N/A
pCrIND:iaaM	This Paper	N/A
pCrIND:iaaL	This Paper	N/A
pCrIND:CrIND:GFP	This Paper	N/A
pAtIND:AtIND:YFP	[40]	N/A
Software and Algorithms		
ImageJ	[41]	https://imagej.nih.gov/ij/
VISTA	[36]	http://genome.lbl.gov/vista

CONTACT FOR REAGENT AND RESOURCE SHARING

Further information and requests for resources and reagents should be directed to and will be fulfilled by the Lead Contact, Lars Østergaard (lars.ostergaard@jic.ac.uk).

EXPERIMENTAL MODEL AND SUBJECT DETAILS

Capsella rubella Cr22.5 and *Arabidopsis thaliana* (Col-0) were used in all experiments of this study. For *Capsella rubella*, the seeds were germinated on MS medium containing 10 μM Gibberellin at 22°C. 10-day-old seedlings were then transplanted into a controlled environment room at 22°C, 16 hr light/8 hr dark conditions. For *Arabidopsis thaliana*, the seeds were germinated on MS medium and 7 days old seedlings were then transplanted to soil and grown in the glasshouse at 22°C, 16 hr light/8 hr dark conditions.

METHOD DETAILS

Plasmids construction and plant transformation

For the construction of the promoter:GUS reporter plasmids of *CrIND*, *CrTAA1* and *CrYUC9*, ~2.0kb promoter was isolated by PCR on genomic DNA and inserted upstream of the *GUS* gene of pCambia1301 vectors. For construction of the *pDR5v2:GFP/GUS* plasmid, a 207-bp promoter fragment containing eight repeats of the auxin response element and 47-bp CaMV 35S minimal promoter [24] was inserted upstream of the GFP and GUS reporter genes of pCambia1301 and pCambia1302 vectors, respectively. For construction of the *pCrIND:CrIND:GFP* plasmid, a ~2.6kb genomic fragment of *CrIND* containing the ~2.0kb promoter and full length coding sequence of *CrIND* was isolated and fused in-frame with the GFP coding sequence of pCambia1302 vector. For construction of the *pCrIND:iaaL/iaaM* plasmids, ~2.0kb *CrIND* promoter was isolated and fused with the full length of the *iaaL/iaaM* coding sequence and then inserted into the pCambia1301 vector. For construction of the RNA-guided genome editing plasmids, DNA sequences encoding gRNAs adjacent to the PAM sequences (NGG) were designed to target two specific sites in the exons of *CrIND*, *CrTAA1* and *CrYUC9*. The gRNAs were synthesized as oligonucleotides with Golden-gate cloning adapters and were then inserted downstream of U6 promoters. The resulting gRNA plasmids were then recombined with *pRPS5a:Cas9z:E9t* and Fast-Red selection marker using golden-gate cloning methods to produce the binary vectors. All vectors were verified by sequencing and introduced into *Agrobacterium tumefaciens* strain LBA4404 by electroporation. Primers used in the construction of the vectors are listed in Methods S1.

Transformation of *Arabidopsis* and *Capsella* followed the floral dipping method with minor modifications in *Capsella*. Specifically, the *Agrobacterium* was cultured to 2.0 (OD_{600}) and resuspended with 5% sucrose solution plus 0.02% Silwet. *Capsella* seedlings with 10cm long inflorescences were subjected to the first round of dipping, after which, the plants were kept in the dark for 36 hours at 22°C. The floral dipping process was repeated twice in five-day intervals. For each construct, at least 10 independent transformants were obtained for further analysis.

Phenotyping and Microscopy

The mature fruits of each genotype were collected and recorded photographically with Nikon D610 camera with a 105mm prime lens.

To quantify the shoulder phenotype, three parameters were measured: W (denotes the half width of the fruits), L1 (denotes the length of the fruit from the fruit shoulder tips to the fruit base) and L2 (denotes the length of the fruit from the style base to the fruit base). The angle of the shoulders was calculated with the anti-trigonometric function $\theta = \mathrm{Arctan}((L1\text{-}L2)/W)$.

For Scanning Electron Microscopy (SEM), the young inflorescences and mature fruits of each genotype were fixed in FAA and infiltrated under vacuum. Gynoecia from distinct developmental stages were dissected with a needle in 70% ethanol under a light

microscope. The materials were critically-point dried in CO_2 and spotter-coated with gold. The samples were subsequently examined using a Zeiss Supra 55VP field Scanning Electron Microscope with an acceleration voltage of 3.0 kV.

Confocal microscopy was performed on a Leica SP5 laser scanning microscope equipped with an Argon Krypton laser (Leica Microsystems). The 488-nm excitation line of an argon ion laser was used to excite GFP, the 514-nm excitation line of an argon ion laser was used to excite YFP. GFP/YFP emission spectra were collected between 497 and 551 nm. For the top views of the gynoecium, the samples were dissected and placed vertically on a slide, we used the X25/0.95 water dipping objective lens to visualize the GFP signal of the specimens. Images were processed in ImageJ software.

Chemical Treatment and Auxin Metabolite Quantification

The auxin biosynthesis inhibitors *L*-Kynurenine and Yucasin were dissolved in DMSO, the Indole-3-acetic acid (IAA) was dissolved in ethanol. For *L*-Kynurenine and Yucasin treatment, 100 μM working solutions were prepared with water and silwet (0.02%) and dipped onto the 10-cm inflorescences. For IAA application, 100 μM working solutions were prepared with water and silwet (0.02%) and applied specifically to the apical part of fruits from WT or *crind-1^{ge} Capsella* plants using a needle. The control plants were mock-treated with the same concentration of either Dimethyl sulfoxide (DMSO) or ethanol used to dissolve the chemicals.

To quantify auxin metabolite levels in the fruit shoulders, WT and *crind-1^{ge}* fruits were dissected under a light microscope. Extraction, purification and the LC-MS/MS analysis of endogenous IAA and specific IAA metabolites was carried out according to the method described previously [42]. Briefly, around 20 mg of frozen material per sample was homogenized and extracted in 1 mL of 50 mM sodium phosphate buffer containing 1% sodium diethyldithiocarbamate and a mixture of $^{13}C_6$ or deuterium labeled internal standards. After centrifugation (14,000 RPM, 15 min, 4°C), the supernatant was divided in two aliquots, the first was derivatised by cysteamine (0.25 M, pH 8, 1h, room temperature, Sigma-Aldrich), the second one was immediately further processed as following. The pH of sample was adjusted to 2.5 by 1 M HCl and the sample was applied on a preconditioned solid-phase extraction column (Oasis HLB, 30 mg, 1 cc, Waters Inc., USA). After sample application, the column was rinsed with 2 mL 5% methanol. Compounds of interest were then eluted with 2 mL 80% methanol. The derivatised fraction was purified alike. Mass spectrometry quantification was performed by LC-MS/MS, using a 1290 Infinity Binary LC System coupled to a 6490 Triple Quad LC/MS System with Jet Stream and Dual Ion Funnel technologies (Agilent Technologies, USA).

RNA extraction and expression analysis

The fruit shoulders and basal fruit were sampled from stage-13 fruits of *Cr*WT and *crind-1^{ge}*, respectively. For the *crful-1, crful-1 crind-1^{ge}* mutants, the whole stage-13 fruits were collected. Total RNA was isolated from the samples using the RNeasy Plant Mini Kit (QIAGEN). Next, 1 ug of total RNA was reverse transcribed into cDNA with the SuperScript IV First-Strand Synthesis System (ThermoFisher) according to the manufacturer's instructions.

For real-time qPCR, gene specific primers were designed, and verified by PCR and sequencing. The efficiency of the primers (95% to 105%) was determined by creating a standard curve. The SYBR Green JumpStart Taq ReadyMix (Sigma-Aldrich) was used to perform real-time qPCR with ROX as a reference dye on a BioRad CFX96 Q-PCR System (BioRad). The CT value of each gene was determined by normalizing the fluorescence threshold. The relative expression level of the target gene was determined using the ratio = $2^{-\Delta CT}$ method, and *CrUBQ10* was used as an internal control. Statistical analysis was done in Microsoft Excel.

For GUS histochemical assay, fruit samples were fixed in acetone for 20 min at −80°C, washed twice for 5 min in 100 mM sodium phosphate buffer, and processed in 100 mM sodium phosphate buffer containing 1mM $K_3Fe(CN)_6$, 1 mM $K_4Fe(CN)_6$ at room temperature for 30 min. The staining was incubated at 37°C in the X-Gluc solution for 6-8h. The X-Gluc solution contains 100 mM sodium phosphate buffer, 10 mM EDTA, 0.5 mM $K_3Fe(CN)_6$, 3 mM $K_4Fe(CN)_6$, 0.1% Triton X-100 and 1 mg/mL of β-glucoronidase substrate X-gluc (5-bromo-4-chloro-3-indolyl glucuronide, MELFORD) dissolved in DMSO. After staining, the reaction buffer was replaced with 70% ethanol until chlorophyll was completely washed out from the samples. Fruits were dissected, mounted in Chlorohydrate (Sigma) solution and analyzed using a Zeiss Axio Imager light microscope.

Chromatin immunoprecipitation and Yeast one-hybrid analysis

Stage-16 fruits from *pCrIND:CrIND:GFP* and WT plants were collected and fixed with 1% formaldehyde and immediately frozen in liquid nitrogen. Approximately 3.0 g of tissue was ground in liquid nitrogen and chromatin fragments were prepared after sonication. After sonication, a 1/20 sample was taken out as DNA Input. The remaining samples underwent immunoprecipitation. GFP tagged protein together with the associated DNAs were immunoprecipitated by using Pierce Protein G Magnetic Beads (ThermoFisher) coated with monoclonal anti-GFP antibody (Roche) according to the manufacturer's instructions. Beads were washed two times with the immunoprecipitation buffer followed by two washes with TE buffer. Reverse crosslinking was done by boiling the beads at 65°C for 12 hours in presence of 10% SDS followed by Proteinase K treatment at 50°C for 1 hour. DNA was ethanol precipitated following phenol/chloroform extraction. qPCR was performed using SYBR Green JumpStart Taq ReadyMix (Sigma-Aldrich) on a BioRad CFX96 Q-PCR System (BioRad).

To perform yeast-one-hybrid (Y1H) analysis, the full length *CrIND* coding sequence was amplified and inserted into the pDEST22 vector (used as the effector plasmid). Synthetic fragments were produced (Sigma) containing wild-type and mutant versions of the putative CrIND binding sites from *CrTAA1*, *CrYUC9* and *CrPID* promoter repeated four times separated by 8-bp spacers. The sequences were then amplified by PCR and inserted into the pHISLEU vector (used as the reporter plasmid). The constructs were co-transformed into the yeast strain, AH109, by using the LiAc method following the instructions for the yeast transformation.

The yeasts transformants were selected on synthetic defined SD/–Trp/–Leu (–WL) agar medium plates and cultured at 28°C. Twelve individual transformants were randomly selected and mixed by three in four Eppendorf tubes, dropped on SD/–Trp/–Leu/–His (–WLH) agar plates and grow at 28°C for 2-3 days to test the interactions. Different concentrations of 3-aminotriazole (3-AT) was applied on the plate to prevent the unspecific interactions.

QUANTIFICATION AND STATISTICAL ANALYSIS

All statistics were calculated in Microsoft Excel. All measured data are presented as means ± SD specified along with sample sizes (n) in the methods and in figure legends. Comparisons between groups for the analysis of qRT-PCR was performed with Microsoft Excel Student's t test, and significance levels are marked as: * $p < 0.05$, ** $p < 0.01$.

Current Biology, Volume *29*

Supplemental Information

Regulatory Diversification of *INDEHISCENT* in the *Capsella* Genus Directs Variation in Fruit Morphology

Yang Dong, Friederike Jantzen, Nicola Stacey, Łukasz Łangowski, Laila Moubayidin, Jan Šimura, Karin Ljung, and Lars Østergaard

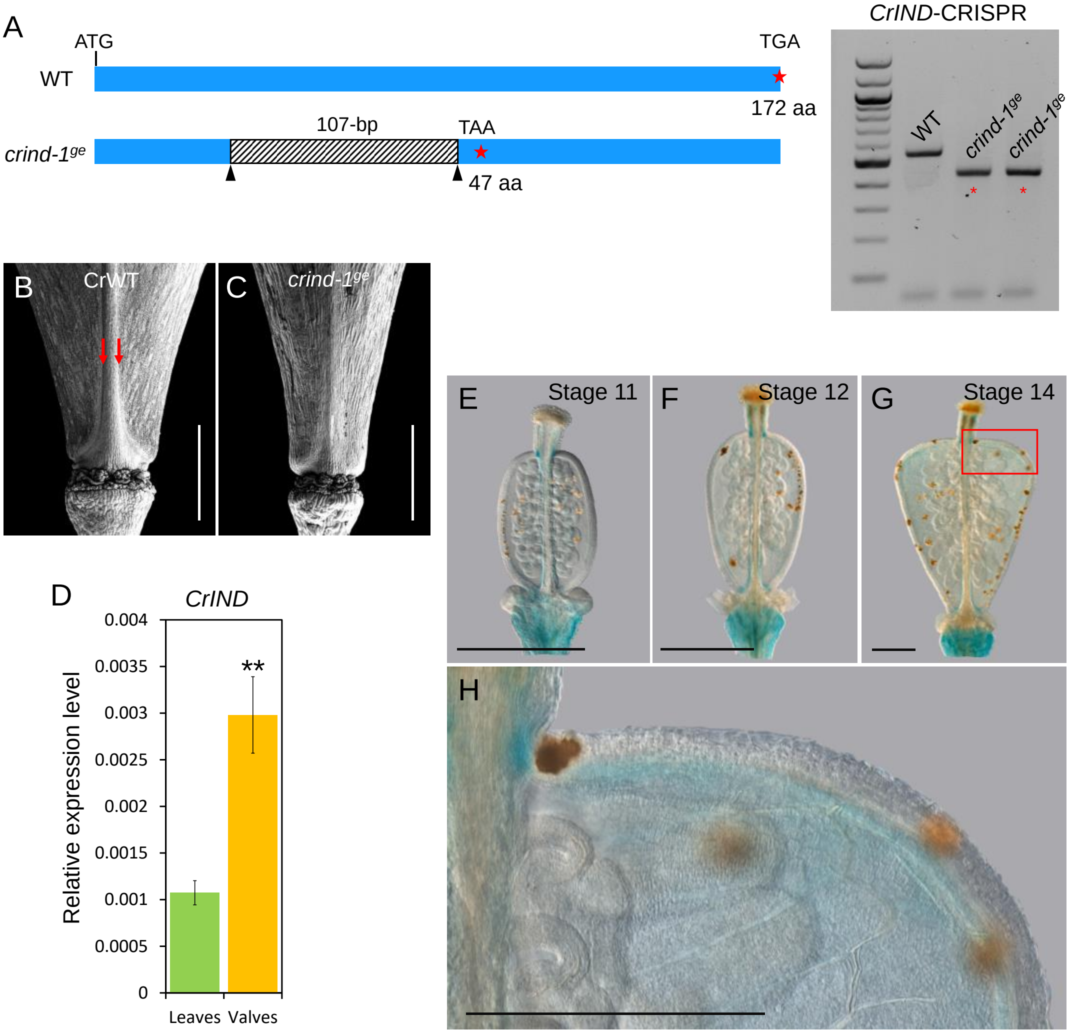

Figure S1. Genotyping of *CrIND* CRISPR line and expression analysis of *CrIND*, Related to Figure 1.

(A) Genotyping of *CrIND* CRISPR lines identified an allele with 107-bp deletion which generate a premature stop codon generating a truncated protein of 47 amino acids in length. (B and C) SEM pictures of basal part of the fruit of CrWT (B) and *crind-1*^{ge} showing the development of valve margins wild type (red arrows). Scale bars, 400 µm. (D) Comparative gene expression analysis of *CrIND* between leaves and fruit valves. Error bars represent SD of three biological replicates. **p<0.01 (Student's *t*-test). (E-H) Expression pattern of *CrIND* during fruit development with *pCrIND:GUS* line. (H) show the enlarged picture of the region outlined with red box in (G). Scale bars, 100 µm.

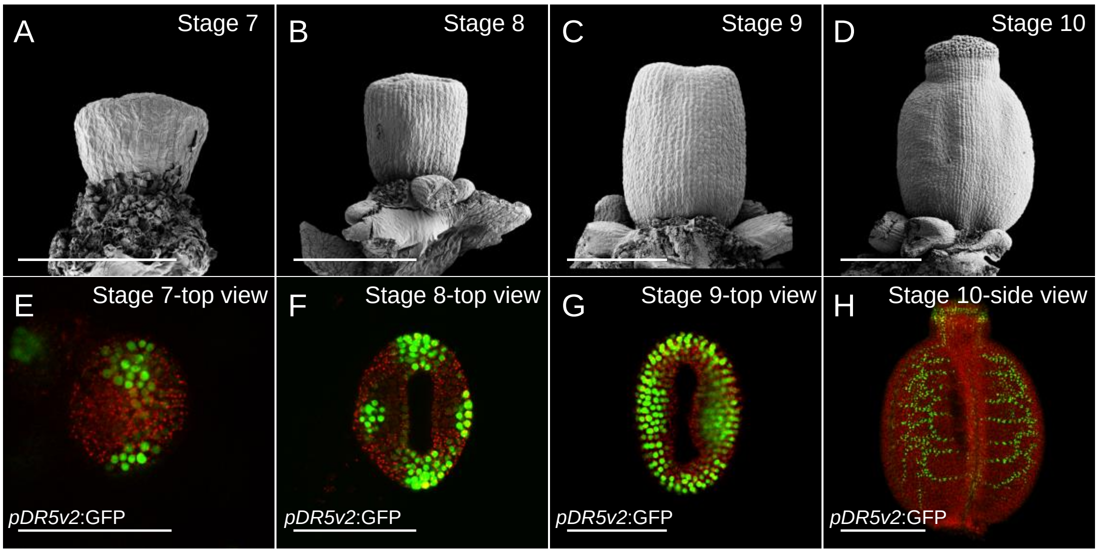

Figure S2. Ontogeny and auxin signaling dynamic during early gynoecium development in *Capsella*, Related to Figure 2.

(A-D) Ontogenetic analysis of the gynoecium of different developmental stages. (E-H) Auxin signaling in gynoecia of different developmental stages as shown by *pDR5v2:GFP*. Scale bars (A-H), 100 μm.

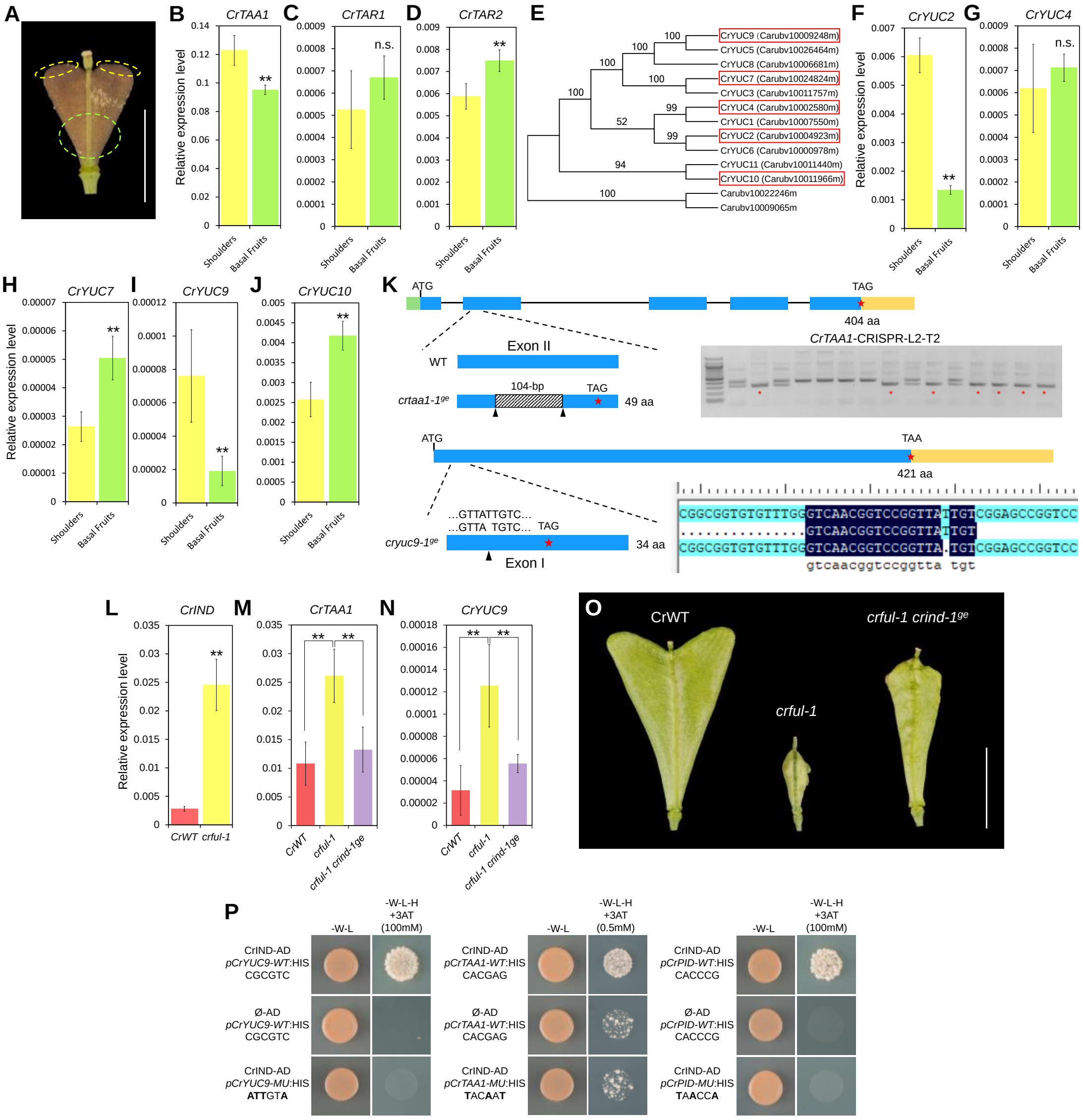

Figure S3. Gene expression analysis of the candidate genes and relationship between *CrIND* and auxin biosynthesis genes, Related to Figure 3.

(A) Graphic view of the fruit tissues sampled for expression analysis. Scale bar, 2 mm. (B-D) Gene expression analysis of the three *Capsella TAA1/TAR* genes compared between tissues sampled as shown in panel A. (E) Neighbor-joining tree of proteins encoded by the 11 *YUC* genes from the *Capsella* genome, bootstrap values over 50% (1,000 replicates) are indicated for each branch. (F-J) Gene expression analysis of five genes belonging to the *YUCCA* family compared between tissues sampled as shown in panel A. (K) Genotyping of the *CrTAA1* and *CrYUC9* CRISPR mutants identified a *crtaa1-1^{ge}* allele with 104-bp deletion in the second exon and a *cryuc9-1^{ge}* allele with a one-base pair deletion in the first exon. (L) Expression analysis of *CrIND* in the whole fruit of CrWT and *crful-1*. (M) and (N) Expression analysis of *CrTAA1* (M) and *CrYUC9* (N) in the whole fruit of CrWT, *crful-1* and *crful-1 crind-1^{ge}* stage-17 fruits. (O) Whole-mount images of fruits from CrWT, *crful-1* and *crful-1 crind-1^{ge}* at stage 17. Scale bar, 5 mm. (P) Yeast-one-hybrid analysis of the interaction of CrIND protein with the variant E box found in the promoters of *CrTAA1* and *CrYUC9*. *CrPID* was used as a positive control. Error bars in (B-D, F-J, L-N) represent SD of three biological replicates. ***p*<0.01 (Student's *t*-test).

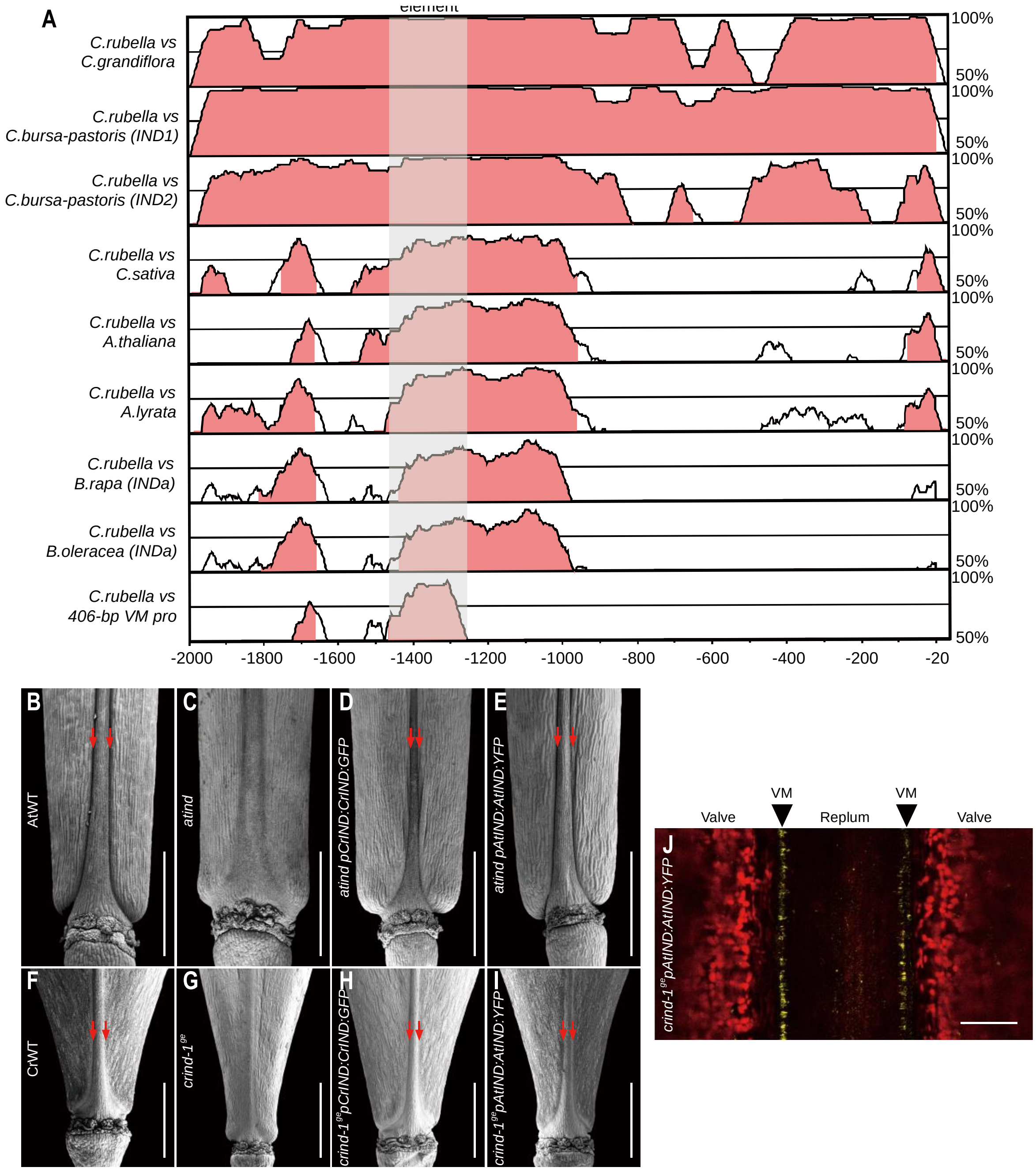

Figure S4. Regulatory divergence in the *CrIND* promoter explains the expression expansion of *CrIND* from the valve margin into the valves, Related to Figure 4.

(A) Phylogenetic shadowing using mVISTA of a ~2.1 kb promoter region upstream of the translational start site of the *IND* gene with pairwise alignments of sequences from *Capsella rubella* with *C. grandiflora, C. bursa-pastoris* (two paralogues), *Camelina sativa*, *Arabidopsis thaliana*, *A. lyrata*, *Brassica rapa*, *B. oleracea* and the 406-bp valve margin element identified previously. Position of the Valve Margin element (VM element) is indicated by a shaded area. (B-E) SEM images of basal fruit of AtWT (B), *atind* (C), *atind pCrIND:CrIND:GFP* (D) and *atind pAtIND:AtIND:YFP* (E). Red arrows in (B), (D) and (E) indicate the valve margin. (F-I) SEM images of basal fruit (8DPA) of CrWT (F), *crind-1*^{ge} (G), *crind-1*^{ge} *pCrIND:CrIND:GFP* (H), *crind-1*^{ge} *pAtIND:AtIND:CFP* (I). (J) Confocal images of fruits of *crind-1*^{ge} *pAtIND:AtIND:YFP* , YFP expression was only detected in the valve margin (VM). Red arrows in (F), (H) and (I) indicate the valve margin. Scale bars, 100 μm (J), 400 μm (B-I).
